# Deciphering the role of immune cell composition in epigenetic age acceleration: Insights from cell‐type deconvolution applied to human blood epigenetic clocks

**DOI:** 10.1111/acel.14071

**Published:** 2023-12-25

**Authors:** Ze Zhang, Samuel R. Reynolds, Hannah G. Stolrow, Ji‐Qing Chen, Brock C. Christensen, Lucas A. Salas

**Affiliations:** ^1^ Department of Epidemiology Geisel School of Medicine at Dartmouth Lebanon New Hampshire USA; ^2^ Dartmouth Cancer Center Dartmouth‐Hitchcock Medical Center Lebanon New Hampshire USA; ^3^ Quantitative Biomedical Sciences Program Guarini School of Graduate and Advanced Studies Hanover New Hampshire USA; ^4^ Molecular and Cellular Biology Program Guarini School of Graduate and Advanced Studies Hanover New Hampshire USA

**Keywords:** biological aging, cell deconvolution, DNA methylation, epigenetic aging, epigenetic clock, epigenetics, immune cell

## Abstract

Aging is a significant risk factor for various human disorders, and DNA methylation clocks have emerged as powerful tools for estimating biological age and predicting health‐related outcomes. Methylation data from blood DNA has been a focus of more recently developed DNA methylation clocks. However, the impact of immune cell composition on epigenetic age acceleration (EAA) remains unclear as only some clocks incorporate partial cell type composition information when analyzing EAA. We investigated associations of 12 immune cell types measured by cell‐type deconvolution with EAA predicted by six widely‐used DNA methylation clocks in data from >10,000 blood samples. We observed significant associations of immune cell composition with EAA for all six clocks tested. Across the clocks, nine or more of the 12 cell types tested exhibited significant associations with EAA. Higher memory lymphocyte subtype proportions were associated with increased EAA, and naïve lymphocyte subtypes were associated with decreased EAA. To demonstrate the potential confounding of EAA by immune cell composition, we applied EAA in rheumatoid arthritis. Our research maps immune cell type contributions to EAA in human blood and offers opportunities to adjust for immune cell composition in EAA studies to a significantly more granular level. Understanding associations of EAA with immune profiles has implications for the interpretation of epigenetic age and its relevance in aging and disease research. Our detailed map of immune cell type contributions serves as a resource for studies utilizing epigenetic clocks across diverse research fields, including aging‐related diseases, precision medicine, and therapeutic interventions.

## Graphical abstract

DNA methylation‐based epigenetic clocks in combination with the advanced 12‐immune‐cell‐deconvolution in human blood offers new opportunities to study intrinsic epigenetic age acceleration and insights into biological implications of immune profile in epigenetic age acceleration in human health and disease.

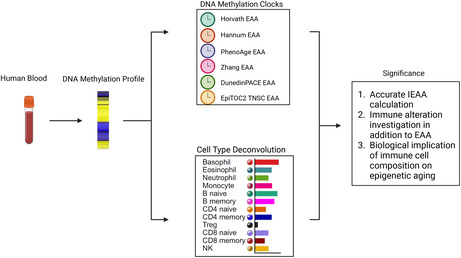

**Abbreviations**
Bas: basophil
Bnv: B naïve cell
Bmem: B memory cell
CD4nv: CD4T naïve cell
CD4mem: CD4T memory cell
CD8nv: CD8T naïve cell
CD8mem: CD8T memory
EAA: epigenetic age acceleration
Eos: eosinophil
GEO: Gene Expression Omnibus
mAge: methylation age
Mono: monocyte
Neu: neutrophil
NK: natural killer cell
PRC2: polycomb repressive complex 2
RA: rheumatoid arthritis
Treg: T regulatory cell

## 1 BACKGROUND

Aging is a well‐known risk factor for various human disorders, encompassing cancer, cardiovascular, endocrine, neurodegenerative, and musculoskeletal diseases (Li et al., 2021; Saul & Kosinsky, 2021). While correlated with chronological age, biological age reflects functional aging and exhibits considerable variation within populations. The difference between chronological age and biological age represents a valuable biomarker for disease risk assessment (Christensen, 2019). In normal tissues, aging‐related methylation changes were identified (Christensen et al., 2009; Kwabi‐Addo et al., 2007). In the last decade, DNA methylation clocks have enabled accurate estimation of biological age, lifespan, and disease risk prediction, and application in age‐reversal research and trials (Fahy et al., 2019; Hannum et al., 2013; Horvath, 2013; Petersen et al., 2021). The first‐generation clocks, such as the Horvath and Hannum clocks, accurately predicted chronological age in human blood (Hannum et al., 2013; Horvath, 2013). Subsequently, Zhang et al. improved the performance of predicting chronological age in human blood by increasing the training sample size (Zhang et al., 2019). The recent wave of second‐generation clock development focused on predicting phenotypic aging by incorporating clinical indicators. Those clocks performed better with health‐related outcomes such as all‐cause mortality and disease risk. For instance, PhenoAge and GrimAge are designed in blood samples to incorporate clinical measures such as immune health and organ‐functional biomarkers for better predictions of lifespan and health span (Levine et al., 2018; Lu et al., 2019). The DunedinPACE blood clock employed longitudinal indicators of organ system integrity to predict the aging pace (Belsky et al., 2022). EpiTOC2 is a mitotic clock that estimates stem cell division rate and is proven to predict cancer risk (Teschendorff, 2020). Prior studies have indicated that epigenetic clocks exhibit tissue‐specific characteristics and perform optimally in their respective tissues due to heterogeneity in cellular composition (Bell et al., 2019; Shireby et al., 2020). Although the Horvath clock was designed as a pan‐tissue clock, it demonstrates the highest accuracy in the blood due to the predominant use of blood samples for training (Horvath, 2013). Additionally, tissue‐specific clocks have been developed for the human brain (Shireby et al., 2020), saliva (Bocklandt et al., 2011), and skeletal muscle (Voisin et al., 2020).

DNA methylation is essential to establishing and preserving cellular identity (Bogdanovic & Lister, 2017). Besides an aging biomarker, it can be used to assess underlying cell‐type proportions in heterogeneous mixtures when combined with a cell‐type reference library (Titus et al., 2017). In recent years, DNA methylation has been widely utilized as a biomarker of different cell types to infer cellular composition. The approach known as DNA methylation deconvolution (or methylation cytometry) offers a standardized and cost‐effective method for evaluating cell‐type proportions (Wiencke, 2020). This technique can be readily employed on preserved samples, making it highly deployable. High‐resolution cell‐type deconvolution has been achieved in various human samples, including blood (Salas et al., 2022), brain (Guintivano et al., 2013; Zhang, Wiencke, et al., 2023), tumor microenvironment (Zhang et al., 2022), skin (Muse et al., 2022), breast biospecimens (Muse et al., 2023), and buccal swabs (Zheng et al., 2018). Specifically in human blood, our previous research employed differentially methylated regions identified between purified leukocyte subtypes to develop a reference‐based deconvolution algorithm, allowing estimation of the distribution of various leukocyte subtypes (Salas et al., 2022). This blood deconvolution approach has been utilized to investigate altered immune cell composition in multiple diseases, such as cancer (Chen et al., 2022, 2023), hypertension (Kresovich et al., 2023), and trisomy 21 (Zhang, Stolrow, et al., 2023).

The underlying biology of epigenetic clocks and their relation with health outcomes remains an important area of investigation with implications for healthy aging and possibly age‐reversal research. Horvath proposed the theory of an epigenetic maintenance system in which epigenetic clocks measure the cumulative work required to maintain epigenetic stability (Horvath, 2013). Recent work studying biological mechanisms underlying epigenetic clocks described the intrinsic and extrinsic components within the clocks (Bell et al., 2019; Chen et al., 2016; Smith et al., 2019). The intrinsic component captures epigenetic aging regulation at the cellular level, emphasizing the potential mechanisms involved in maintaining the epigenome during aging. For instance, Polycomb Repressive Complex 2 (PRC2) targets are enriched in the aging‐related CpGs and play a crucial role in regulating aging‐related gene expression (Cao et al., 2021; Dozmorov, 2015). The PRC2 epigenomic signature is associated with hypermethylation and gene expression changes in aging while stable across cell types (Dozmorov, 2015). On the other hand, the extrinsic component focused on the aging effect that alters cell composition within a tissue. Specifically in human blood, T cell, and natural killer (NK) cell activation were reported as drivers of epigenetic clock progression (Jonkman et al., 2022). Epigenetic age acceleration (EAA), derived from the comparison between epigenetic age and chronological age, is often studied as an indicator of health‐related outcomes (Chilunga et al., 2021; Faul et al., 2023; Jain et al., 2022; Monasso et al., 2021). To disentangle the intrinsic and extrinsic aging effects within the epigenetic clocks, previous research employed multivariable models to adjust for cell counts or proportions when studying EAA with the outcomes of interest (Chen et al., 2016; Zhang et al., 2019). However, the extent to which cell composition impacts epigenetic clocks remains unknown. With advancements in high‐resolution cell‐type deconvolution based on DNA methylation in blood (Salas et al., 2022), we systematically investigated the association between immune cell composition and EAA predicted using popular DNA methylation clocks.

## 2 RESULTS

**FIGURE 1:**
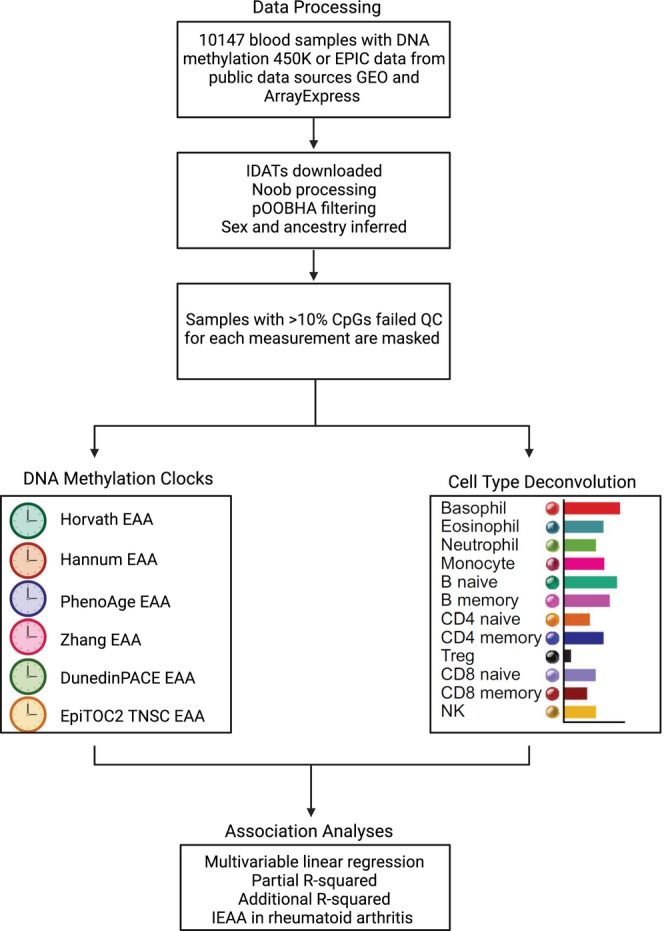
Flowchart of the study design.

A total of 10,147 blood samples with DNA methylation data from publicly accessible sources were included in this study (Table 1). Figure S1 illustrates the distribution of ages among the subjects. A flowchart summarizing the study is presented in Figure 1.

**TABLE 1:** Characteristics of data sets.

	No reported disease	Diseased
*N*	6223	3924
Demographics		
Age (Mean (SD))	50.57 (22.34)	53.09 (19.45)
InferredSex = Male (%)	2024 (32.5)	1749 (44.6)
InferredAncestry (%)		
East Asian	37 (0.6)	1 (0.02)
African (Subsaharan)	1681 (27.0)	18 (0.5)
European	4505 (72.4)	3905 (99.5)
Disease (%)		
No reported disease	6223 (100)	–
Bronchopulmonary dysplasia	–	14 (0.4)
COVID‐19	–	407 (10.4)
Depression	–	489 (12.5)
Down syndrome (Trisomy 21)	–	17 (0.4)
Gestational stress or smoking	–	27 (0.7)
Gestational stress and smoking	–	11 (0.3)
Gestational diabetes mellitus	–	165 (4.2)
Non‐muscle‐invasive bladder cancer	–	601 (15.3)
Paget's disease of bone	–	232 (5.9)
Parkinson's disease	–	334 (8.5)
Polybrominated biphenyl exposure	–	679 (17.3)
Periodontitis	–	480 (12.2)
Rheumatoid arthritis	–	354 (9.0)
Sporadic Creutzfeldt‐Jakob disease	–	114 (2.9)
Epigenetic Clock		
Horvath age (Mean (SD))	51.59 (20.30)	55.04 (19.37)
Hannum age (Mean (SD))	46.40 (25.63)	55.65 (22.84)
PhenoAge (Mean (SD))	43.50 (26.82)	45.37 (27.21)
ZhangEN age (Mean (SD))	47.73 (24.44)	51.93 (25.35)
DunedinPACE (Mean (SD))	1.06 (0.15)	1.02 (0.14)
EpiTOC2_TNSC (Mean (SD))	3435.02 (1353.08)	3144.83 (1152.93)
Epigenetic Age Acceleration		
Horvath age acceleration (Mean (SD))	1.04 (6.80)	1.74 (6.44)
Hannum acceleration (Mean (SD))	1.40 (6.17)	0.86 (6.83)
PhenoAge acceleration (Mean (SD))	−6.83 (10.27)	−7.97 (12.32)
ZhangEN age acceleration (Mean (SD))	−1.57 (5.41)	−1.89 (8.06)
Cell‐type Deconvolution		
Basophil (Mean (SD))	1.30 (3.01)	0.96 (2.44)
B memory cell (Mean (SD))	1.35 (1.71)	1.53 (2.42)
B naive cell (Mean (SD))	4.49 (4.02)	3.01 (2.62)
CD4T memory cell (Mean (SD))	7.19 (5.36)	6.77 (5.22)
CD4T niave cell (Mean (SD))	5.34 (5.22)	4.56 (4.94)
CD8T memory cell (Mean (SD))	7.94 (5.86)	5.77 (5.75)
CD8T naive cell (Mean (SD))	1.31 (2.06)	1.02 (1.68)
Eosinophil (Mean (SD))	1.32 (2.28)	1.49 (2.15)
Monocyte (Mean (SD))	8.04 (2.51)	7.50 (2.82)
Neutrophil (Mean (SD))	54.34 (14.45)	59.92 (13.09)
Natural killer cell (Mean (SD))	4.61 (2.50)	4.29 (2.47)
T regulatory cell (Mean (SD))	1.28 (1.69)	0.97 (1.58)

### 2.1 Association between EAA and immune cell composition

**FIGURE 2:**
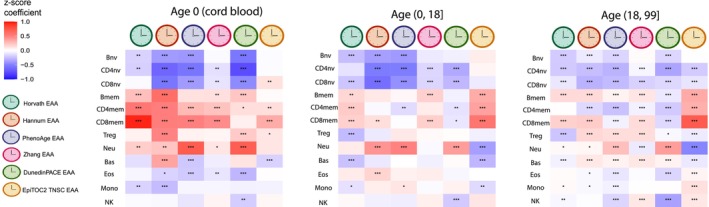
The association between immune cell composition and EAA for Horvath, Hannum, Pheno, and Zhang, DunedinPACE, and EpiTOC2 TNSC clocks across three age groups (0, 0–18, 18–99). Sex, chronological age, ancestry, and disease status were adjusted (*FDR < 0.05, **FDR < 0.01, ***FDR < 0.001).

The association between immune cell composition and EAA after adjusting for chronological age, sex, ancestry, and disease status is summarized in Figure 2. Consistent across the age groups, the distinction between naive and memory lymphocyte compartments emerges as a pivotal factor influencing EAA. In newborns, memory lymphocyte proportions (including Bmem, CD4mem, and CD8mem) consistently displayed a positive correlation with EAA across six distinct clocks, while the naive states of B, CD4T, and CD8T lymphocyte proportions showed a negative association with EAA in five out of six clocks. In the population aged between 0 and 18, naive lymphocyte proportions (Bnv, CD4nv, and CD8nv) consistently exhibited negative associations with EAA in five out of six clocks. The influence of the lymphocyte subpopulations on EAA varied in adult populations. Naive lymphocyte proportions (Bnv, CD4nv, and CD8nv) consistently displayed negative associations with EAA across six clocks. Bmem cell proportions showed a significant negative association with EAA in the DunedinPACE clock and significant positive associations with EAA in all other clocks. CD4mem cell proportions demonstrated significant negative associations with EAA in Hannum, PhenoAge, Zhang, and DunedinPACE clocks while positively associated with EAA in the EpiTOC2 TNSC clock. CD8mem cell proportions exhibited significant positive associations with EAA in Horvath, Hannum, Zhang, and EpiTOC2 TNSC clocks while displaying significant negative associations with EAA in PhenoAge and DunedinPACE clocks. Other immune cell types also exhibited significant associations with EAA across different clocks, with varying directions, particularly Neu and Treg. This comprehensive analysis offers valuable insights into potential immune mechanisms influencing EAA. In a sensitivity analysis, we used the difference between methylation age (mAge) and chronological age as EAA to investigate the association between EAA and cell type proportions in all populations. The results are consistent with the EAA calculated using the residuals, demonstrated in Figure S2. Scatterplots illustrating the correlations between EAA and immune cell proportions are provided in Figures S3–S8.Figure S9 demonstrates the associations between individual immune cells and EAA while accounting for the influence of other immune cell proportions.

### 2.2 Advances in intrinsic epigenetic age acceleration (IEAA) calculation

**FIGURE 3:**
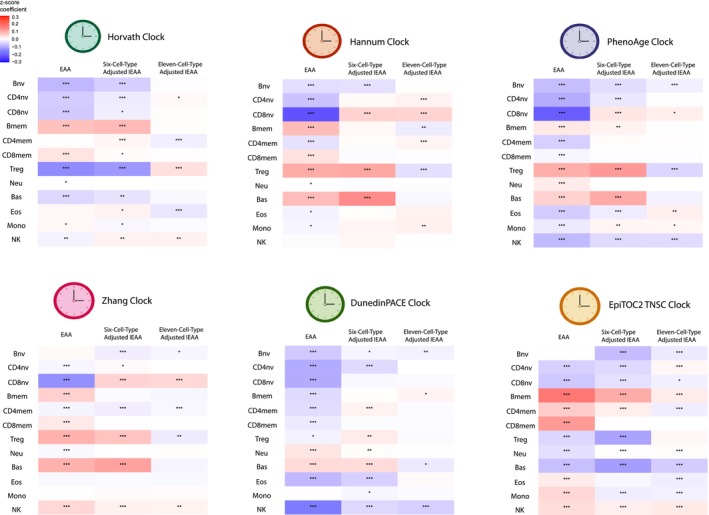
The association between immune cell composition and EAA and IEAA for Horvath, Hannum, PhenoAge, Zhang, DunedinPACE, and EpiTOC2 TNSC clocks. Sex, chronological age, ancestry, and disease status were adjusted (*FDR <0.05, **FDR <0.01, ***FDR <0.001).

Using the 12‐immune‐cell‐deconvolution method, more immune cell types, especially the naïve and memory subtypes, can be adjusted to calculate IEAA. The 11‐cell‐type‐adjusted IEAA showed the weakest association with cell type proportions compared to the unadjusted EAA and traditional six‐cell‐type‐adjusted IEAA across all clocks, demonstrated in Figure 3, indicating the necessity of employing the more granular deconvolution method to adjust for cell types in IEAA calculations. The findings offer new opportunities to study IEAA in human disease and health more accurately.

### 2.3 EAA partial R‐squared by chronological age, immune cell composition, sex, ancestry, and disease status

**FIGURE 4:**
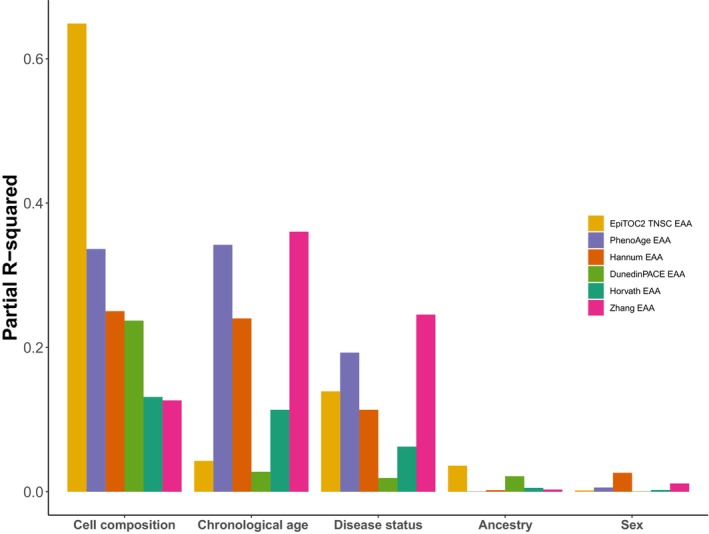
The proportion of EAA variation that was explained by chronological age, immune cell composition, disease status, sex, and ancestry, respectively reflected by partial R‐squared across the six clocks.

Figure 4 illustrates the proportion of EAA variation explained by different variables, including immune cell composition, chronological age, disease status, sex, and ancestry. Immune cell composition has varying contributions to the variation of EAA. Immune cell composition emerges as the most influential factor for EpiTOC2 TNSC EAA (Partial *R* ^2^ = 0.65), Hannum EAA (Partial *R* ^2^ = 0.25), DunedinPACE EAA (Partial *R* ^2^ = 0.24), and Horvath EAA (Partial *R* ^2^ = 0.13), while being second highest in PhenoAge EAA (Partial *R* ^2^ = 0.336), slight below chronological age (Partial *R* ^2^ = 0.34). Zhang EAA showed higher explanations by chronological age (Partial *R* ^2^ = 0.36) and disease status (Partial *R* ^2^ = 0.25), compared to immune cell composition (Partial *R* ^2^ = 0.13). Sex and ancestry contribute minimally to mAge variation (Partial *R* ^2^ < 0.04).

### 2.4 Additional R‐squared added by the immune cell types

**FIGURE 5:**
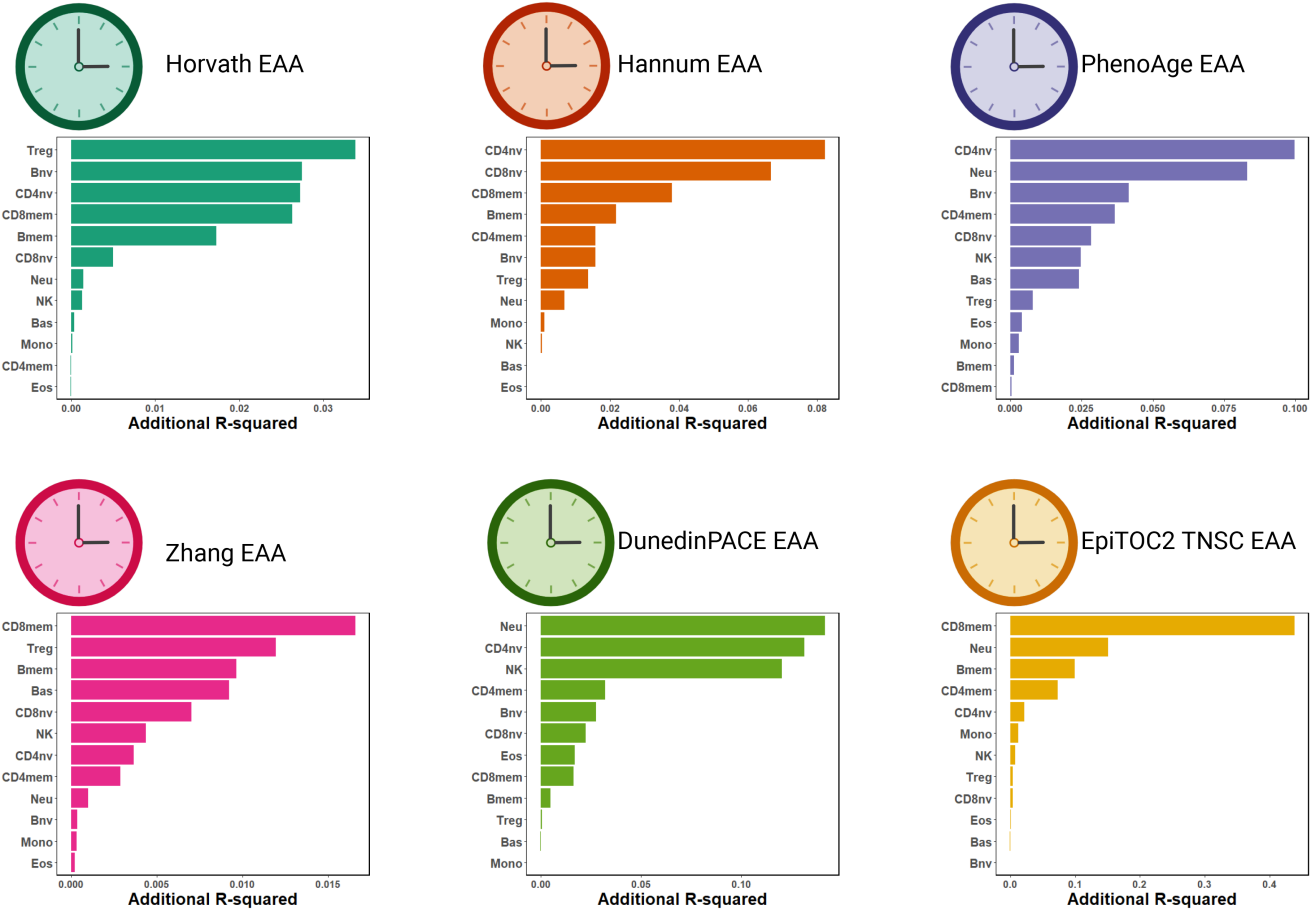
The unique variance that each immune cell type added to explain the EAA variation in addition to chronological age, sex, disease status, and ancestry reflected by additional R‐squared across the six clocks.

Figure 5 summarizes the additional unique variance contributed by each immune cell type in explaining EAA variation in addition to chronological age, sex, disease status, and ancestry. For the Horvath EAA, the top five significant immune cell types are Treg, Bnv, CD4nv, CD8mem, and Bmem. The Hannum EAA shows CD4nv, CD8nv, CD8mem, Bmem, and CD4mem as the top five significant immune cell types. CD4nv, Neu, Bnv, CD4mem, and CD8nv emerge as the top five significant immune cell types for the PhenoAge EAA. The Zhang EAA demonstrates CD8mem, Treg, Bmem, Bas, and CD8nv as the top five significant immune cell types. The DunedinPACE EAA indicates Neu, CD4nv, NK, CD4mem, and Bnv as the top five significant immune cell types. For the EpiTOC2 TNSC EAA, the top five significant immune cell types are CD8mem, Neu, Bmem, CD4mem, and CD4nv. Overall, the naïve and memory compartments of lymphocytes play a significant role in explaining EAA variation.

### 2.5 EAA in rheumatoid arthritis (RA) with immune cell composition adjusted

**FIGURE 6:**
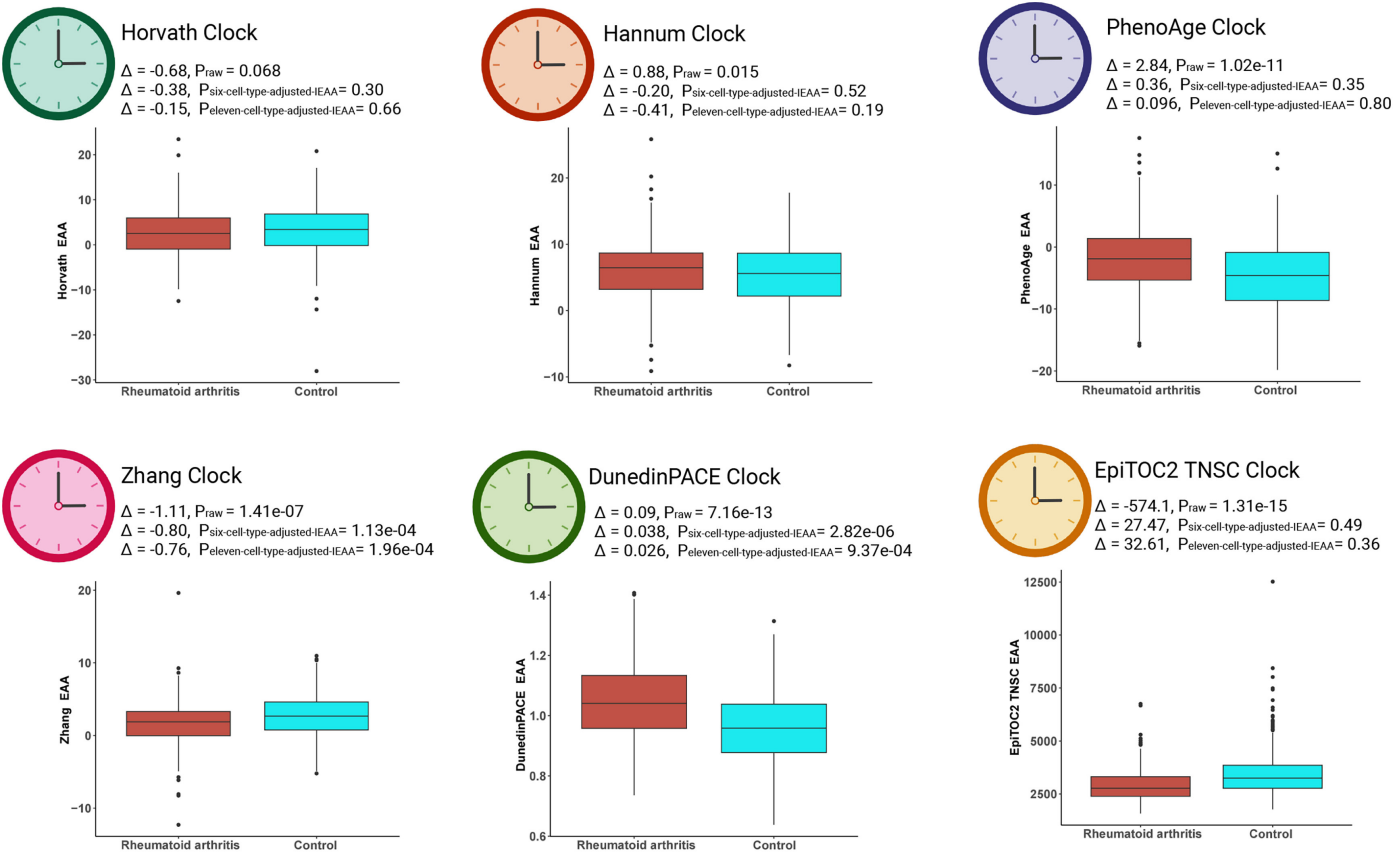
The comparisons of EAA and IEAA derived from Horvath, Hannum, PhenoAge, Zhang, DunedinPACE, and EpiTOC2 TNSC clocks between RA cases and controls.

We compared the results from multiple variable linear regression models to investigate EAA change in RA cases compared to controls with and without adjustment of immune cell composition (Figure 6). The EAA derived from the Horvath clock did not show significant differences between RA cases and controls before and after immune cell composition adjustments (*p* > 0.05). For the Hannum and PhenoAge clocks, we initially observed significant increases in EAA among RA cases compared to controls before adjusting for immune cell composition (Hannum EAA: *p*_raw_ = 0.015; PhenoAge EAA: *p*_raw_ = 1.02e−11). However, these differences became non‐significant after adjusting for immune cell composition (Hannum IEAA: *p*_six‐cell‐type‐adjusted−IEAA_ = 0.52, *p*_eleven‐cell‐type‐adjusted−IEAA_ = 0.19; PhenoAge IEAA: *p*_six‐cell‐type‐adjusted‐IEAA_ = 0.35, *p*_eleven‐cell‐type‐adjusted‐IEAA_ = 0.80). The EAA calculated from the Zhang clock showed significant decreases in RA cases compared to controls before and after immune cell composition adjustments (*p*_raw_ = 1.4e−07; *p*_six‐cell‐type‐adjusted‐IEAA_ = 1.1e−04; *p*_eleven‐cell‐type‐adjusted‐IEAA_ = 2e−04). The DunedinPACE clock showed significantly increased aging in RA cases and controls before and after immune cell composition adjustments (*p*_raw_ = 7.2e−13; *p*_six‐cell‐type‐adjusted‐IEAA_ = 2.8e−06; *p*_eleven‐cell‐type‐adjusted‐IEAA_ = 9.4e−04). The EpiTOC2 TNSC clock showed significant decreases in stem cell mitotic division rate in RA cases compared to controls before adjusting for immune cell composition (*p*_raw_ = 1.3e−15). However, the difference was non‐significant after adjusting for immune cell composition (*p*_six‐cell‐type‐adjusted‐IEAA_ = 0.49, *p*_eleven‐cell‐type‐adjusted‐IEAA_ = 0.36). Additionally, we identified significant increases in Neu and Treg cell populations, along with decreases in Bmem, Bnv, CD4mem, CD4nv, CD8mem, CD8nv, Mono, and NK cell populations in RA cases compared to controls (Figure S10).

### 2.6 Methylation clocks in purified immune cells

We evaluated the performance of Horvath, Hannum, PhenoAge, and Zhang clocks in predicting chronological age using 12 purified immune cell types. The results revealed limited performance of these clocks on purified immune cell types, particularly within the naïve and memory compartments of lymphocytes (Figure S11). The DunedinePACE clock only tracks chronological age significantly in Mono (Pearson's *r* = 0.96, *p* = 0.01, Figure S12). The EpiTOC2 clock only tracks chronological age significantly in CD4nv (Pearson's *r* = 0.96, *p* = 0.01, Figure S13). The distributions of EAA in purified cell types are shown in Figures S14–S19, respectively, for Horvath, Hannum, PhenoAge, Zhang, DunedinPACE, and EpiTOC2 TNSC clocks. EAAs were consistently lower in CD8nv compared to their memory counterparts CD8mem, across all six clocks. Lower EAAs were observed in CD4nv compared to CD4mem in five out of six clocks. Bnv showed lower levels of EAA compared to Bmem in PhenoAge, Zhang, and EpiTOC2 TNSC clocks. CD8nv exhibited the lowest EAA levels in Horvath, Hannum, Zhang, PhenoAge, and DunedinPACE clocks, while granulocytes showed the lowest EAA levels in the EpiTOC2 TNSC clock. Eos showed the highest EAA levels for both Horvath and Hannum clocks. Mono showed the highest EAA level for PhenoAge and DunedinPACE clocks, whereas Bmem showed the highest EAA level for Zhang and EpiTOC2 TNSC clocks.

### 2.7 Pediatric clock EAA and immune cell composition in non‐adult populations

The associations between immune cell composition and pediatric clock EAA after adjusting for chronological age, sex, ancestry, and disease status are summarized in Figure S20. Within the 0–5 age group, significant positive associations were observed between FCO EAA and Bnv, CD4nv, and Bas, while a negative association was observed with Neu. In the 0–18 age group, the Wu EAA demonstrated significant positive associations with CD4mem and CD8mem and negative associations with Bnv, CD4nv, and CD8nv.

## 3 DISCUSSION

DNA methylation‐based epigenetic clocks have emerged as valuable tools for tracing chronological age and predicting various health‐related outcomes such as mortality and disease risk (Bell et al., 2019). These clocks have also been crucial biomarkers in anti‐aging research and age‐reversal trials (Fahy et al., 2019; Fitzgerald et al., 2021). However, despite their widespread use, epigenetic clocks' mechanisms and interpretations remain incompletely understood, mainly due to the diverse methods employed in developing different clocks. In our study, we systematically investigated the relationship between immune cell composition and EAA in human blood, utilizing DNA methylation‐based cell‐type deconvolution along with popular epigenetic clocks. To our knowledge, our research represents the first comprehensive mapping of immune cell type contributions to mainstream epigenetic clock variations.

The immune cell composition was found to be a significant factor influencing the variation of Horvath, Hannum, and PhenoAge clocks, ranking second after chronological age. Notably, the Zhang clock, which aimed to improve age prediction accuracy by including large cohorts of elderly individuals without disease status information (Zhang et al., 2019), resulted in disease status becoming the second most important variable explaining the clock's variation, followed by immune cell composition. Unlike the epigenetic clocks that directly predict chronological age, DunedinePACE and EpiTOC2 TNSC clocks measure biological aging pace and mitotic stem cell division rate. Both clocks demonstrated immune cell composition as the most significant variable contributing to their variation. Particularly, the EpiTOC2 TNSC clock showed the highest explanation of clock variation by immune cell composition among all the tested clocks.

While a previous study conducted by Bozack et al. (2023) established associations between EAA and six immune cell types in blood, it did not distinguish between the naive and memory compartments of lymphocytes. Our findings revealed that the naïve and memory subsets of lymphocytes played a crucial role in explaining the variation of EAA. The memory cell compartment positively drove EAA, while the naïve cell compartment exerted a negative influence. The specific naïve and memory cell types contributing significantly to EAA varied across clocks. These observations align with previous reports highlighting T‐cell activation as a driver of epigenetic clock progression (Jonkman et al., 2022). Decreases in naïve cell numbers and increases in memory cell numbers related to age have been well‐documented in previous studies (Lazuardi et al., 2005; Li et al., 2019; Salam et al., 2013). Our results indicate that epigenetic clocks rely substantially on such features to trace chronological age. CD8mem is the major driver of the mitotic stem cell division rate measured by the EpiTOC2 TNSC clock, which could be explained by the observation that memory T cells initiate cell division more rapidly than their naïve counterparts (Whitmire et al., 2008) and CD8 T cells divide faster and show a greater level of clonal expansion than CD4 T cells (Kaech et al., 2002). Our research has established a connection between different lymphocyte states and EAA, implicating the interplay between intrinsic and extrinsic components of epigenetic aging clocks. One potential mechanism is through the aging regulation of PRC2. In previous studies, PRC2 targets were established to be enriched in the aging‐related CpGs and associated with hypermethylation and gene expression changes caused by aging effect (Guintivano et al., 2013). PRC2 also plays a critical role in maintaining cellular identity and controlling cell fate decisions. EZH2 proteins, the core catalytic subunit of PRC2, are essential to regulate T cell development, differentiation, and function (Huang, Zhang, et al., 2021; Stairiker et al., 2020). In aging, several studies have suggested that PRC2 activity and the levels of H3K27me3 can be altered, leading to changes in gene expression patterns (Dozmorov, 2015; Moqri et al., 2022; Tauc et al., 2021). We posit that intrinsic aging effects impact PRC2 activity at the cellular level, causing compositional changes in lymphocytes, specifically transitioning from naïve to memory states. While prior research has differentiated intrinsic and extrinsic aging mechanisms of epigenetic clocks, we argue that they may not be mutually exclusive biologically. Both components are essential for developing an accurate epigenetic clock, as they are intertwined. Thus, one cannot be completely differentiated from the other. Neu emerged as a major positive driving force of EAA in myeloid cells. Indeed, Neu is the only positive driver of DunedinPACE's aging pace. Li et al. reported a dynamic trend of Neu proportion in whole blood before adulthood (Macallan et al., 2019). The Neu proportion experienced a steep drop within the first 6 months of age and showed an increasing trend until 18 years of age. Salas et al. also showed an increase in Neu proportion until adulthood and stabilized afterward. The aging effect on the Neu proportion is a potential positive contributor to EAA and the aging pace. Initially, we observed a negative association between Bas proportion and EAA. However, we found that the high Bas levels largely influenced this observation in cord blood. After removing newborn populations from the analysis, the negative association turned positive in three out of four clocks. Nucleated red blood cells (nRBCs) are rare in adult blood but are present in cord blood. Our initial immune cell deconvolution study showed that nRBCs cluster with Bas using the markers in the deconvolution (Salas et al., 2022). Thus, we hypothesize that the original negative association between Bas proportion and EAA is attributed to the higher levels of nRBCs in cord blood.

Epigenetic age acceleration was observed to be associated with various disease outcomes, including cancer, cardiovascular disease, neurodegenerative diseases, metabolic disorders, and infections (Baldelli et al., 2023; Dong et al., 2022; Horvath & Levine, 2015; Liu et al., 2022; McCartney et al., 2018; Monasso et al., 2021; Nannini et al., 2019). Chen et al. showed EAA derived from Hannum and PhenoAge clocks was associated with worse 10‐year overall survival rates in bladder cancer patients. They also showed that multiple immune cell types were significantly associated with 10‐year overall survival rates. Notably, CD8nv, a major negative driver of EAA, initially correlated with better survival rates but switched to a worse prognosis after adjusting for EAA, indicating CD8nv as a potential confounding factor in the association between EAA and survival. EAA was observed in blood samples from populations with trisomy 21 (Horvath et al., 2015; Xu et al., 2022). At the same time, consistent decreases in naïve lymphocytes and increases in memory lymphocytes were observed in trisomy 21 patients (Zhang, Stolrow, et al., 2023). The direction of EAA and naïve and memory lymphocyte change suggest the immune cell composition's confounding effect on EAA in trisomy 21. Pang et al. (2022) demonstrated that COVID‐19 patients exhibit elevated PhenoAge and GrimAge, which they partially attributed to alterations in immune cell composition. Our study highlighted the confounding effect of immune cell composition on EAA in RA. In RA patients, the Neu proportion represents the largest cell type discrepancy when compared with control samples. Hannum and PhenoAge age acceleration in RA was initially observed but non‐significant after adjusting for immune cell composition. The Neu proportion, as a positive driver in EAA, can contribute substantially as a confounding factor to the observed EAA in RA. Furthermore, the decrease of mitotic stem cell division rate measured by EpiTOC2 TNSC in RA is likely to be confounded by the decrease of CD8mem proportion in RA. We recommend incorporating immune cell composition into the EAA analysis in future studies. The cell type proportions should be adjusted to study the biological aging effect on outcomes without being confounded by the immune cell composition, particularly in the naïve and memory compartments of lymphocytes and neutrophils. Furthermore, we recommend using DNA methylation‐based cell type deconvolution to directly study immune cell composition change when studying EAA, as both methods are feasible with DNA methylation data.

Epigenetic clocks have become valuable tools in anti‐aging research and age‐reversal trials (Fahy et al., 2019; Fitzgerald et al., 2021). When aiming to achieve age reversal, researchers should consider whether the reversal is at the cellular or cell composition levels. As epigenetic clocks rely significantly on the aging effect on cellular composition, the implications of biological age measured by epigenetic clocks matter at both the cellular and bulk tissue levels. Therefore, adjusting for cell composition is crucial when measuring epigenetic aging in age‐reversal studies conducted in bulk tissues such as whole blood, and assessing both cellular composition and epigenetic aging allows for a comprehensive depiction of anti‐aging effects at both the cellular and bulk levels. Prior research has made strides in distinguishing between intrinsic and extrinsic epigenetic acceleration when studying EAA in the context of aging (Chen et al., 2016; McGuire, 1971; Smith et al., 2019). The utilization of the 12‐immune‐cell‐type deconvolution method offers an opportunity for a more precise differentiation between IEAA and EEAA. For IEAA, this advanced method allows for calculating IEAA at a significantly finer level of granularity than was previously feasible. While earlier technologies were restricted to adjusting IEAA for up to seven immune cell types, our approach extends this to an unprecedented 11 cell types, considerably reducing the confounding effects of immune cell composition. Regarding EEAA, conventional methods calculate it by assigning weights to limited immune cell types within epigenetic clocks. Although these approaches augment the contribution of immune cell composition to EAA to some extent, they fail to disentangle the effects of intrinsic and extrinsic aging, making interpretation challenging. To enhance the accuracy of EEAA calculations, in future studies, we propose to train a model to predict biological age using CpGs known to be cell‐type‐specific, such as those from the immune cell deconvolution library. In summary, we recommend adopting the 12‐immune‐cell‐type deconvolution method for IEAA calculation and for comparing differences in immune cell composition within specific conditions of interest in aging research. This additional approach provides valuable insights into the direct impact of the exposure of interest on the immune system, a facet that EAA only partially reflects.

While our study comprehensively described the associations between EAA and immune cell composition across mainstream epigenetic clocks, several limitations should be acknowledged. First, cell proportions were estimated using DNA methylation‐based cell type deconvolution, necessitating the validation of findings with whole blood counts to establish the association between cell counts and EAA. Second, although our study focused on blood, cell composition and EAA vary across different tissue types (Horvath, 2013). Future studies should expand the analysis to include more tissues, ideally using tissue‐specific epigenetic clocks and deconvolution methods, such as those applicable to brain tissue (Shireby et al., 2020; Zhang, Wiencke, et al., 2023). Third, our study included limited subjects in the adolescent age range. Additional cohorts with subjects aged 5–20 years could enhance the analysis. Fourth, although our study includes six mainstream epigenetic clocks, it is important to note that numerous other epigenetic clocks exist, such as GrimAge, another popular clock used for predicting health‐related outcomes (Lu et al., 2019). However, the specific CpGs used in the GrimAge clock are not publicly accessible, making it unfeasible for inclusion in our quality control pipeline. As a result, our analysis was limited to the clocks for which the necessary data were available. Future studies should aim to incorporate additional epigenetic clocks to provide a more comprehensive evaluation of epigenetic age acceleration and its associations with immune cell composition. Fifth, we acknowledge the limited sample size for examining epigenetic clocks in purified immune cells. Despite consistently observing lower performance in specific cell types and marked differentiation in EAA across various purified cell types, future studies with an increased sample size are necessary to enhance statistical power and confirm the findings. Lastly, while the current deconvolution methods included certain types of naïve and memory lymphocytes (B, CD4T, and CD8T), the inclusion of additional types, such as NK and Treg lymphocytes, would contribute to a better understanding of lymphocyte activation in EAA.

## 4 CONCLUSION

By employing DNA methylation‐based cell‐type deconvolution and epigenetic clocks, our study introduces several critical advancements in the field of epigenetic aging research. Utilizing an advanced 12‐cell‐type immune cell deconvolution method, we achieved IEAA calculation with unprecedented granularity, significantly reducing cell composition and confounding effects. Moreover, we emphasize the importance of examining immune cell composition within the context of conditions of interest, providing a more comprehensive understanding of factors of interest directly impact the immune system beyond what EAA can reveal. Finally, our research has unveiled the significant contributions of various immune cell subsets to epigenetic aging, enriching our understanding of the intricate relationship between immune cell composition and aging. These findings hold promise for aging‐related research, precision medicine, and therapeutic interventions, and we encourage researchers to adopt these insights into their future studies on EAA.

## 5 METHODS

### 5.1 Data sets

In this study, we utilized publicly available datasets from the Gene Expression Omnibus (GEO) and ArrayExpress. These datasets consisted of Illumina methylation 450 K or EPIC bead array IDAT files, comprising a comprehensive collection of 10,147 samples (Table 1) (Arloth et al., 2020; Castro de Moura et al., 2021; Chen et al., 2022; Chuang et al., 2017; Curtis et al., 2019; Dabin et al., 2020; Diboun et al., 2022; Huang, Cai, et al., 2021; Johansson et al., 2013; Kasuga et al., 2022; Kurushima et al., 2019; Liu et al., 2013; Maschietto et al., 2017; Naumova et al., 2021; Perez et al., 2019; Shang et al., 2023; Tan et al., 2014; Van Baak et al., 2018; Wang et al., 2018, 2022). The subjects included in our study spanned an age range of 0–99 years, ensuring a wide representation of age groups.

### 5.2 Data processing

*GEOquery* from *Bioconductor* in R was used to download IDAT files and phenotype data from GEO (Davis & Meltzer, 2007). For ArrayExpress data sets, IDAT files and phenotype data were directly downloaded from the website. The normal‐exponential out‐of‐band (Noob) method from the *Minfi* package was used to process the IDAT files (Aryee et al., 2014). The Noob preprocessing pipeline was recommended for methylation‐based blood deconvolution (Salas et al., 2022). For quality control, *p*‐value with out‐of‐band array hybridization (*p*_OOBHA_) was employed from the *SeSAMe* package (Zhou et al., 2018). *p*_OOBHA_ > 0.05 was used as the cutoff to mask low‐quality probes. All data sets reported chronological age. However, sex and ancestry information were inconsistent across the data sets. DNA methylation on X and Y chromosome probes can be used to infer sex. Also, an ancestry proxy can be inferred using SNPs and channel‐switching Type‐I probes on the microarray. We used the *SeSAMe* package to infer sex and ancestry for all subjects included in this study for consistency.

### 5.3 DNA methylation clocks

Six DNA‐methylation‐based epigenetic clocks were used in this study. Horvath, Hannum, DunedinPACE, and PhenoAge clocks were employed using the *methyAge* function from the *ENMIX* package (Belsky et al., 2022; Hannum et al., 2013; Horvath, 2013; Levine et al., 2018; Xu et al., 2016). The Zhang clock was directly employed using the elastic net predictor from their GitHub webpage (https://github.com/qzhang314/DNAm‐based‐age‐predictor) (Zhang et al., 2019). The estimated cumulative number of stem cell divisions per stem cell per year and per sample was calculated using the *EpiTOC2* software *TNSC2* function from https://zenodo.org/record/2632938#.ZHPDGnbMKUl (Teschendorff, 2020). For non‐adult populations, we utilized the Wu pediatric clock by applying the *DNAmAge* function in the *methylclock* package (Pelegi‐Siso et al., 2021; Wu et al., 2019). The FCO clock was calculated using the CpGs and coefficients provided in the Salas paper (Salas et al., 2018). The libraries of the CpGs for each clock were extracted. For quality control, samples with >10% of CpGs from the clock‐specific library failed *p*_OOBHA_ < 0.05 filtering process and were masked for that specific clock. EAA was defined as residuals from linear regression with methylation age as an outcome and chronological age as a predictor. In a sensitivity analysis, EAA was calculated for Horvath, Hannum, PhenoAge, and Zhang clocks as methylationage−chronologicalage. DunedinPACE and EpiTOC2 TNSC clocks were not reflected by age in years, thus not used for the sensitivity analysis.

### 5.4 DNA methylation‐based immune cell deconvolution

DNA methylation‐based cell type deconvolution method from the *FlowSorted.BloodExtended.EPIC* package was used to infer 12 immune cell proportions in blood samples, including basophils (Bas), eosinophils (Eos), neutrophils (Neu), monocytes (Mono), B naïve cells (Bnv), B memory cells (Bmem), CD4T naïve cells (CD4nv), CD4T memory cells (CD4mem), T regulatory cells (Treg), CD8T naïve cells (CD8nv), CD8T memory cells (CD8mem), and NK cells (Salas et al., 2022). For quality control, samples with >10% of CpGs from the deconvolution library failed pOOBHA < 0.05 filtering process were masked.

### 5.5 Multivariable linear regression

The study population was categorized into three distinct age groups: 0, 0–18, and 18–99 age groups. *Z*‐scores with means set to 0 and standard deviations set to 1 were computed for both EAA and immune cell proportions within each age group for every epigenetic clock, ensuring the comparability of results across all clocks. Multivariable linear regression models were used to study the association between EAA and immune cell proportions for Horvath, Hannum, PhenoAge, Zhang, DunedinPACE and EpiTOC2 TNSC clocks (Equation 1). In addition to adjusting for sex, ancestry, and disease status in the model, chronological age and chronological age‐squared terms were included in the model to account for the nonlinear association between epigenetic clock accuracy and chronological age as suggested by Shireby et al. (Shireby et al., 2020).

(1) $$\begin{array}{cc} \mathrm{EAA}\_{\text{zscore}}_{i}= & \beta_{0}+\beta_{1}\text{CellType}\_{\text{zscore}}_{j}+\beta_{2}\mathrm{Sex}+\beta_{3}\text{Ancestry}\\ & +\beta_{4}\text{Disease}+\beta_{5}\mathrm{Age}+\beta_{6}{\mathrm{Age}}^{2} \end{array}$$

In Equation 1, *i* represents epigenetic clocks (Horvath, Hannum, PhenoAge, Zhang, DunedinPACE, EpiTOC2 TNSC) and *j* represents immune cell types (Bas, Eos, Neu, Mono, Bnv, Bmem, CD4nv, CD4mem, Treg, CD8nv, CD8mem, NK). For each of the i×j models fit, we tested the hypothesis that the mean EAA for clock *i* doesn't change with an increase of the cell *j* proportion ($H_{0}:\beta_{1}=0$). An FDR of 0.05 was used as the statistical significance cutoff threshold. We conducted a sensitivity analysis with same models using differences between methylation age and chronological age as EAA in all population.

Furthermore, we conducted a sensitivity analysis involving non‐adult populations, utilizing two pediatric epigenetic clocks. Specifically, for individuals aged 0–18, we employed the Wu clock, which is specifically designed for estimating biological age in children aged 9–212 months. For those in the age range of 0 to 5, we applied the Fetal Cell Origin (FCO) DNA methylation signature, designed to trace cells of fetal origin and known to inversely track with age before 5 years old. Both Wu and FCO EAA values were calculated using residuals. Subsequently, we calculated *Z*‐scores and employed Equation 1 to investigate the association between EAA and immune cell composition.

### 5.6 Intrinsic epigenetic age acceleration and immune cell composition

Three sets of EAA, including raw EAA, six‐cell‐type‐adjusted IEAA, and eleven‐cell‐type‐adjusted IEAA, were compared with immune cell composition across six clocks. The raw EAA was calculated using residuals without any adjustment for cell type proportions. The six‐cell‐type‐adjusted IEAA was computed by adjusting residuals for the traditional six cell types, including CD8nv, CD8mem, Bcell, Mono, CD4T, and Gran. The eleven‐cell‐type‐adjusted IEAA was calculated using residuals adjusting for eleven cell types, including CD8nv, CD8mem, Bnv, Bmem, Mono, CD4nv, CD4mem, Bas, Eos, Neu, and Treg. *Z* For standardization, *Z*‐scores were calculated for both IEAA and immune cell proportions for each epigenetic clock, with means set to 0 and standard deviations set to 1. We then employed multivariable linear regression models to investigate the associations between IEAA and immune cell proportions using Equation 2.

(2) $$\begin{array}{cc} \text{IEAA}\_{\text{zscore}}_{i}=\; & \beta_{0}+\beta_{1}\text{CellType}\_{\text{zscore}}_{j}+\beta_{2}\mathrm{Sex}+\beta_{3}\text{Ancestry}+\\ & \beta_{4}\text{Disease}+\beta_{5}\mathrm{Age}+\beta_{6}{\mathrm{Age}}^{2} \end{array}$$

In Equation 2, *i* represents epigenetic clocks and *j* represents immune cell types (Bas, Eos, Neu, Mono, Bnv, Bmem, CD4nv, CD4mem, Treg, CD8nv, CD8mem, NK). For each of the i×j models fit, we tested the hypothesis that the mean IEAA for clock *i* doesn't change with an increase of the cell *j* proportion ($H_{0}:\beta_{1}=0$). An FDR of 0.05 was used as the statistical significance cutoff threshold.

### 5.7 Partial R‐squared analysis

To test the proportion of EAA variation explained by chronological age, cell composition, disease status, sex, and ancestry respectively, partial R‐squared was calculated with the full and reduced models using the *rsq.partial* function from the *rsq* package (Zhang, 2017). The full model is shown in Equation 3.

(3) $${\mathrm{EAA}}_{i}=\beta_{0}+\sum_{j=1}^{11}\beta_{j}{\text{CellType}}_{j}+\beta_{12}\mathrm{Sex}+\beta_{13}\text{Ancestry}+\beta_{14}\text{Disease}+\beta_{15}\mathrm{Age}+\beta_{16}{\mathrm{Age}}^{2}$$

The reduced models to test for the proportion of mAge variation explained by chronological age is shown in Equation 4.

(4) $${\mathrm{EAA}}_{i}=\beta_{0}+\sum_{j=1}^{11}\beta_{j}{\text{CellType}}_{j}+\beta_{12}\mathrm{Sex}+\beta_{13}\text{Ancestry}+\beta_{14}\text{Disease}$$

The reduced models to test for the proportion of mAge variation explained by immune cell composition is shown in Equation 5.

(5) $${\mathrm{EAA}}_{i}=\beta_{0}+\beta_{1}\mathrm{Sex}+\beta_{2}\text{Ancestry}+\beta_{3}\text{Disease}+\beta_{4}\mathrm{Age}+\beta_{5}{\mathrm{Age}}^{2}$$

The reduced models to test for the proportion of mAge variation explained by disease status is shown in Equation 6.

(6) $${\mathrm{EAA}}_{i}=\beta_{0}+\sum_{j=1}^{11}\beta_{j}{\text{CellType}}_{j}+\beta_{12}\mathrm{Sex}+\beta_{13}\text{Ancestry}+\beta_{14}\mathrm{Age}+\beta_{15}{\mathrm{Age}}^{2}$$

The reduced models to test for the proportion of mAge variation explained by sex is shown in Equation 7.

(7) $${\mathrm{EAA}}_{i}=\beta_{0}+\sum_{j=1}^{11}\beta_{j}{\text{CellType}}_{j}+\beta_{12}\text{Ancestry}+\beta_{13}\text{Disease}+\beta_{14}\mathrm{Age}+\beta_{15}{\mathrm{Age}}^{2}$$

The reduced models to test for the proportion of mAge variation explained by ancestry is shown in Equation 8.

(8) $${\mathrm{EAA}}_{i}=\beta_{0}+\sum_{j=1}^{11}\beta_{j}{\text{CellType}}_{j}+\beta_{12}\mathrm{Sex}+\beta_{13}\text{Disease}+\beta_{14}\mathrm{Age}+\beta_{15}{\mathrm{Age}}^{2}$$

In Equations (3), (4), (5), (6), (7), (8), *i* represents the six epigenetic clocks and *j* represents 11 immune cell types as we removed NK cell to avoid collinearity in the model. The choice to exclude NK cells, as opposed to other cell types, is a result of the consideration for mitigating issues related to collinearity and maximizing the elimination of cell type confounding effects within the regression models. Based on the immune profile of the samples, there are three cell types that have less than 1% of 0 proportions, i.e., NK, monocyte, and neutrophil. NK is selected because it has a minimal proportion across those three cell types. Although arbitrary to some extent, the approach minimizes the confounding effect by cell type with consideration for collinearity in the models.

### 5.8 Additional R‐squared analysis

To test the unique variance that each immune cell type added to explain the EAA variation in addition to chronological age, sex, disease status, and ancestry, we calculated the change in R‐squared with each immune cell type added to the model. The baseline model is shown in Equation 9 and the immune cell type added model is shown in Equation 10.

(9) $${\mathrm{EAA}}_{i}=\beta_{0}+\beta_{1}\mathrm{Sex}+\beta_{2}\text{Ancestry}+\beta_{3}\text{Disease}+\beta_{4}\mathrm{Age}+\beta_{5}{\mathrm{Age}}^{2}$$

(10) $${\mathrm{EAA}}_{i}=\beta_{0}+\beta_{1}\mathrm{Sex}+\beta_{2}\text{Ancestry}+\beta_{3}\text{Disease}+\beta_{4}\mathrm{Age}+\beta_{5}{\mathrm{Age}}^{2}+\beta_{6}{\text{CellType}}_{j}$$

In Equations 9 and 10, *i* represents the six epigenetic clocks, and *j* represents the 12 immune cell types. The additional R‐squared for each immune cell type for each clock is calculated as$\;\text{Equation}\;10\;R^{2}-\text{Equation}\;9\;R^{2}$.

### 5.9 EAA in RA with immune cell composition adjusted

To test the impact of immune cell composition on EAA in RA, we compared the results from multiple variable linear regression models with and without adjustment of immune cell composition. The data was subset to subjects with RA and without any reported disease as controls. To control for chronological age, sex, and ancestry between the RA and control groups, the *match.it* function from the *MatchIt* package was used to subset the control group subjects to match with the RA group on chronological age, sex, and ancestry (Ho et al., 2011). The information on the subjects included in this analysis is shown in Table S1. Three sets of EAA, including raw EAA, six‐cell‐type‐adjusted IEAA, and eleven‐cell‐type‐adjusted IEAA, were used. The raw EAA was calculated using residuals without any adjustment for cell type proportions. The six‐cell‐type‐adjusted IEAA was computed by adjusting residuals for the traditional six cell types, including CD8nv, CD8mem, Bcell, Mono, CD4T, and Gran. The eleven‐cell‐type‐adjusted IEAA was calculated using residuals adjusting for eleven cell types, including CD8nv, CD8mem, Bnv, Bmem, Mono, CD4nv, CD4mem, Bas, Eos, Neu, and Treg. The models used are shown in Equations 11–14.

(11) $${\mathrm{EAA}}_{i}=\beta_{0}+\beta_{1}\text{RAStatus}$$

(12) $${\text{IEAA}}_{i}=\beta_{0}+\beta_{1}\text{RAStatus}$$

In Equations 11 and 12, *i* represents epigenetic clocks.

### 5.10 mAge and EAA in purified immune cells

To evaluate the performance of the six epigenetic clocks in purified immune cell types, we used the data set from GSE167998 on GEO, which encompasses samples from 12 distinct immune cell types with varying chronological ages (Table S2) (Salas et al., 2022). mAge is regressed against chronological age for the 12 immune cell types, respectively. The predictive performance of the clocks in estimating chronological age was evaluated using RMSE, R‐squared, and p‐value. Additionally, we calculated the EAA for each epigenetic clock in relation to each cell type.

### 5.11 Principal component analysis

A principal component analysis (PCA) was conducted to assess the variation of immune cell composition in the studied population. In the PCA, the relationships between the top principal components (PC1 and PC2) and multiple variables were examined, including data sources (i.e., batch), age, sex, ancestry, and disease. The PCA plot indicated that PC1 and PC2 demonstrated distinct separations by disease and age, while no discernible separation based on data sources was observed (Figure S21). This observation led us to conclude that any potential batch effect is minimal in our study.

### 5.12 Independent association between individual immune cells and EAA

To explore the associations between individual immune cells and EAA while accounting for the influence of other immune cell proportions, we performed a mutually adjusted analysis. This involved simultaneously considering all immune cell proportion *z*‐scores in the same model for each EAA *z*‐score, as depicted in Equation 13.

(13) $$\begin{array}{cc} \mathrm{EAA}\_{\text{zscore}}_{i}=\; & \beta_{0}+\sum_{j=1}^{11}\beta_{j}\text{CellType}\_{\text{zscore}}_{j}+\beta_{12}\mathrm{Sex}+\beta_{13}\text{Ancestry}\\ & +\beta_{14}\text{Disease}+\beta_{15}\mathrm{Age}+\beta_{16}{\mathrm{Age}}^{2} \end{array}$$

In Equation 13, *i* represents epigenetic clocks and *j* represents immune cell types (Bas, Eos, Neu, Mono, Bnv, Bmem, CD4nv, CD4mem, Treg, CD8nv, CD8mem). For each of the i×j models fit, we tested the hypothesis that the mean EAA for clock *i* doesn't change with an increase of the cell *j* proportion, adjusting for other immune cell proportions, ancestry, disease status, and age ($H_{0}:\beta_{j}=0$). An FDR of 0.05 was used as the statistical significance cutoff threshold.

All analyses were performed using R version 4.3.0.

## AUTHOR CONTRIBUTIONS

Z.Z., L.A.S., and B.C.C. conceived the project and designed the studies. Z.Z., S.R.R, H.G.S., and J.Q.C. performed data acquisition, quality control, and statistical analyses. Z.Z. wrote the manuscript with input from all the co‐authors. All authors read and approved the final manuscript.

## FUNDING INFORMATION

This research is supported by the Department of Defense (W81XWH‐20‐1‐0778), the National Institute of General Medical Sciences (P20GM104416/8299, P20GM130454), NCI Division of Cancer Treatment and Diagnosis (R01CA253976), and NCI Division of Cancer Control and Population Sciences (R01CA216265 and P30CA023108).

## CONFLICT OF INTEREST STATEMENT

The authors declare that they have no competing interests.

## CONSENT FOR PUBLICATION

The final version of the manuscript has been reviewed and approved by all authors.

## Supporting information


Appendix S1.

## Data Availability

All data sets used in this study are publicly available on GEO and ArrayExpress. The accession numbers are GSE42861, GSE61496, GSE62219, GSE85042, GSE87571, GSE99863, GSE111629, GSE116339, GSE121633, GSE122086, GSE125105, GSE156994, GSE158063, GSE167998, GSE168739, GSE174555, GSE183920, GSE188949, GSE201322, GSE210256, E‐MTAB‐7069, and E‐MTAB‐7309.
