# Supplementary material for: Deciphering the role of immune cell composition in epigenetic age acceleration: Insights from cell‐type deconvolution applied to human blood epigenetic clocks
Source: Aging Cell. 2023 Dec 25;23(3):e14071. doi: 10.1111/acel.14071 (PMC10928575; doi:10.1111/acel.14071)
Supplement: Supplementary file 1 — Appendix S1. [file ACEL-23-e14071-s001.docx]

Supplementary Materials

**Unveil Epigenetic Clocks with Cell-Type Deconvolution in Human Blood: Implications of Immune Cell Composition on Epigenetic Age Acceleration**

Table of Contents

**Supplementary Figure 1.** The distribution of ages among the subjects included in the study2

**Supplementary Figure 2.** The association between immune cell composition and Horvath EAA, Hannum EAA, PhenoAge EAA, Zhang EAA, DunedinPACE, and EpiTOC2 TNSC ……………………………………………………….3

**Supplementary Figure 3.** The correlations between Horvath EAA and immune cell proportions 4

**Supplementary Figure 4.** The correlations between Hannum EAA and immune cell proportions.5

**Supplementary Figure 5.** The correlations between PhenoAge EAA and immune cell proportions6

**Supplementary Figure 6**. The correlations between Zhang EAA and immune cell proportions7

**Supplementary Figure 7.** The correlations between DunedinPACE acceleration and immune cell proportions8

**Supplementary Figure 8.** The correlations between EpiTOC2 TNSC acceleration and immune cell proportions9

**Supplementary Figure 9.** The associations between individual immune cells and EAA while accounting for the influence of other immune cell proportions10

**Supplementary Figure 10.** Immune cell proportion differences between RA cases and controls 11

**Supplementary Figure 11.** The performance of Horvath, Hannum, PhenoAge, and Zhang clocks in predicting chronological age using 12 purified immune cell types12

**Supplementary Figure 12.** The correlations between DunedinPACE and chronological age in 12 purified immune cell types 13

**Supplementary Figure 13.** The correlations between EpiTOC2 TNSC and chronological age in 12 purified immune cell types 14

**Supplementary Figure 14.** The distribution of Horvath EAA in purified immune cell types15

**Supplementary Figure 15.** The distribution of Hannum EAA in purified immune cell types16

**Supplementary Figure 16.** The distribution of PhenoAge EAA in purified immune cell types 17

**Supplementary Figure 17.** The distribution of Zhang EAA in purified immune cell types 18

**Supplementary Figure 18.** The distribution of DunedinPACE EAA in purified immune cell types19

**Supplementary Figure 19.** The distribution of EpiTOC2 TNSC EAA in purified immune cell types20

**Supplementary Figure 20.** The association between immune cell composition and pediatric clock EAA after adjusting for chronological age, sex, ancestry, and disease status21

**Supplementary Figure 21.** The relationships between the top principal components (PC1 and PC2) and multiple variables were examined, including data sources (i.e., batch), age, sex, ancestry, and disease..22

**Supplementary Table 1.** The summary of sex, age, and ancestry matched RA cases and controls23

**Supplementary Table 2.** Purified immune cell samples 24

**
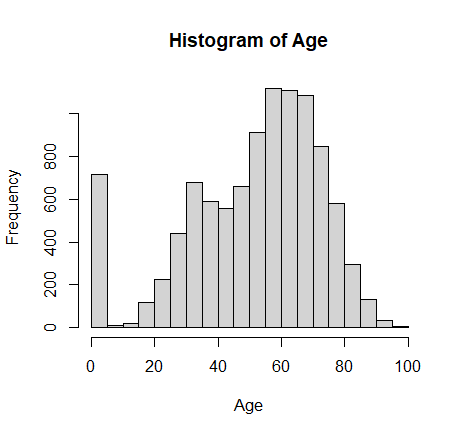
**

**Supplementary Figure 1.** The distribution of ages among the subjects included in the study.


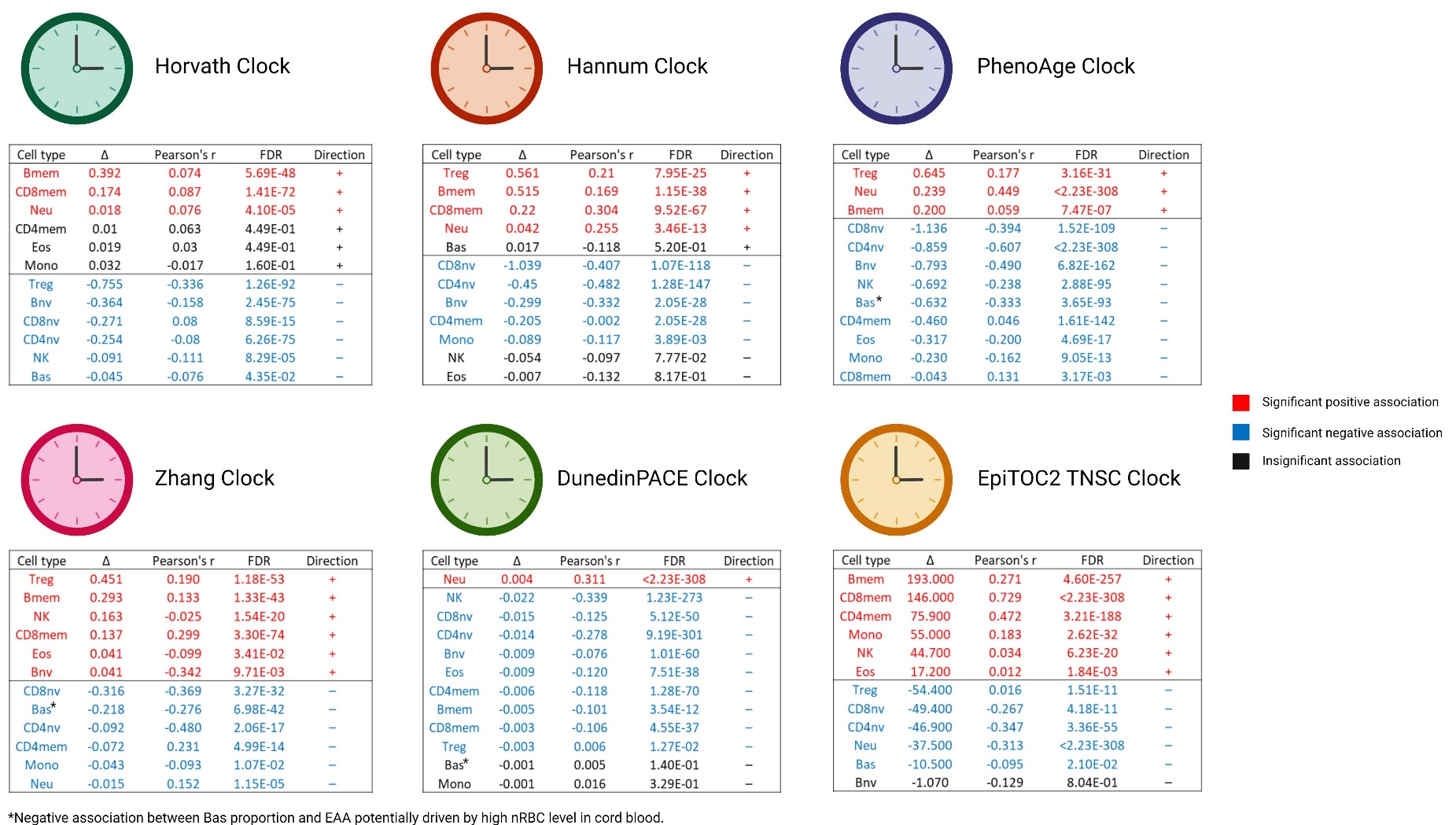


**Supplementary Figure 2.** The association between immune cell composition and Horvath EAA, Hannum EAA, PhenoAge EAA, Zhang EAA, DunedinPACE aging pace, and EpiTOC2 TNSC mitotic stem-cell division rate. Δ represents the change in EAA, DunedinPACE aging pace, and EpiTOC2 TNSC, with a 1% increase in the proportion of the corresponding immune cell. Sex, age, ancestry, and disease status were adjusted. EAA was calculated using the difference between the chronological age and epigenetic age.


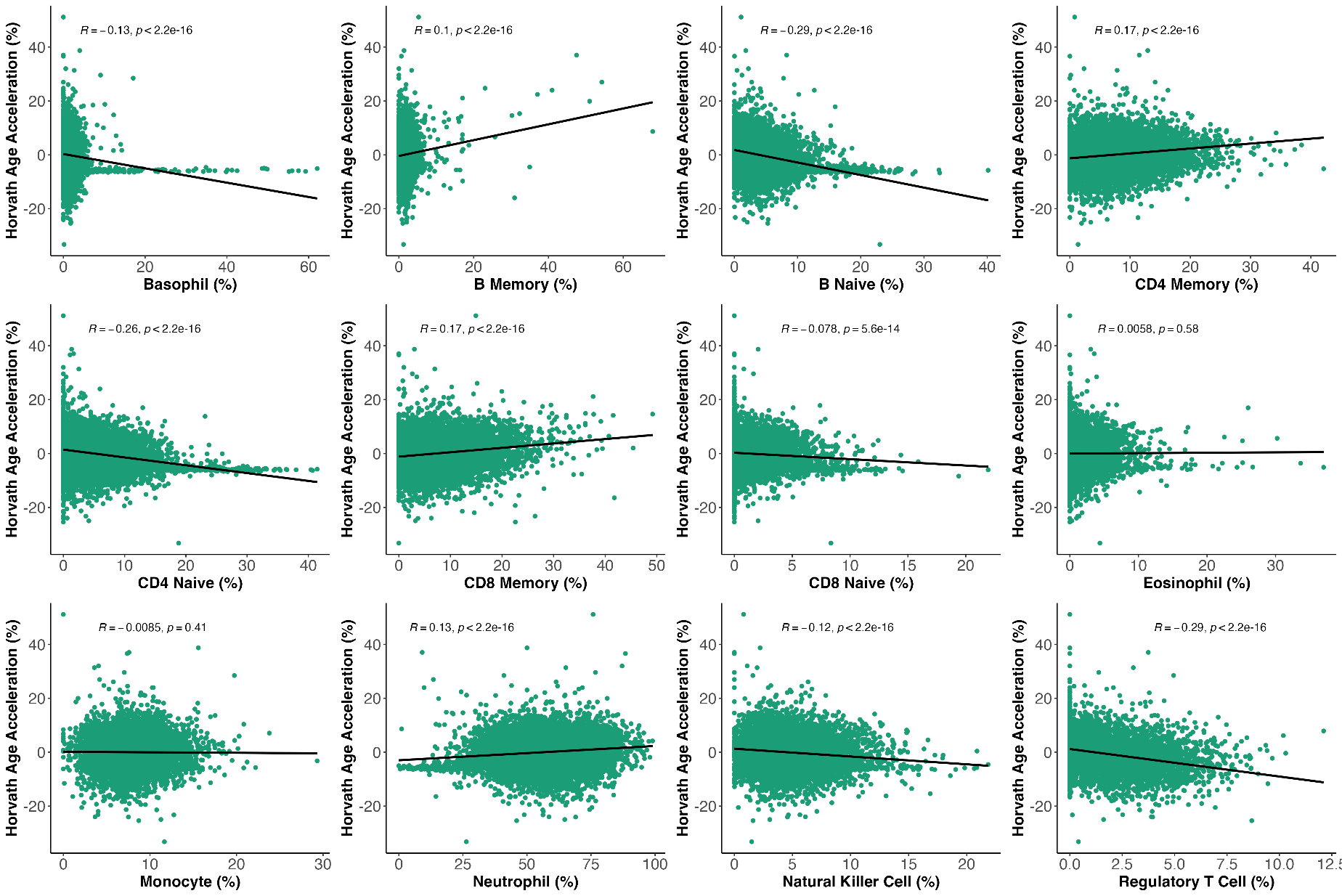


**Supplementary Figure 3.** The correlations between Horvath EAA and immune cell proportions.


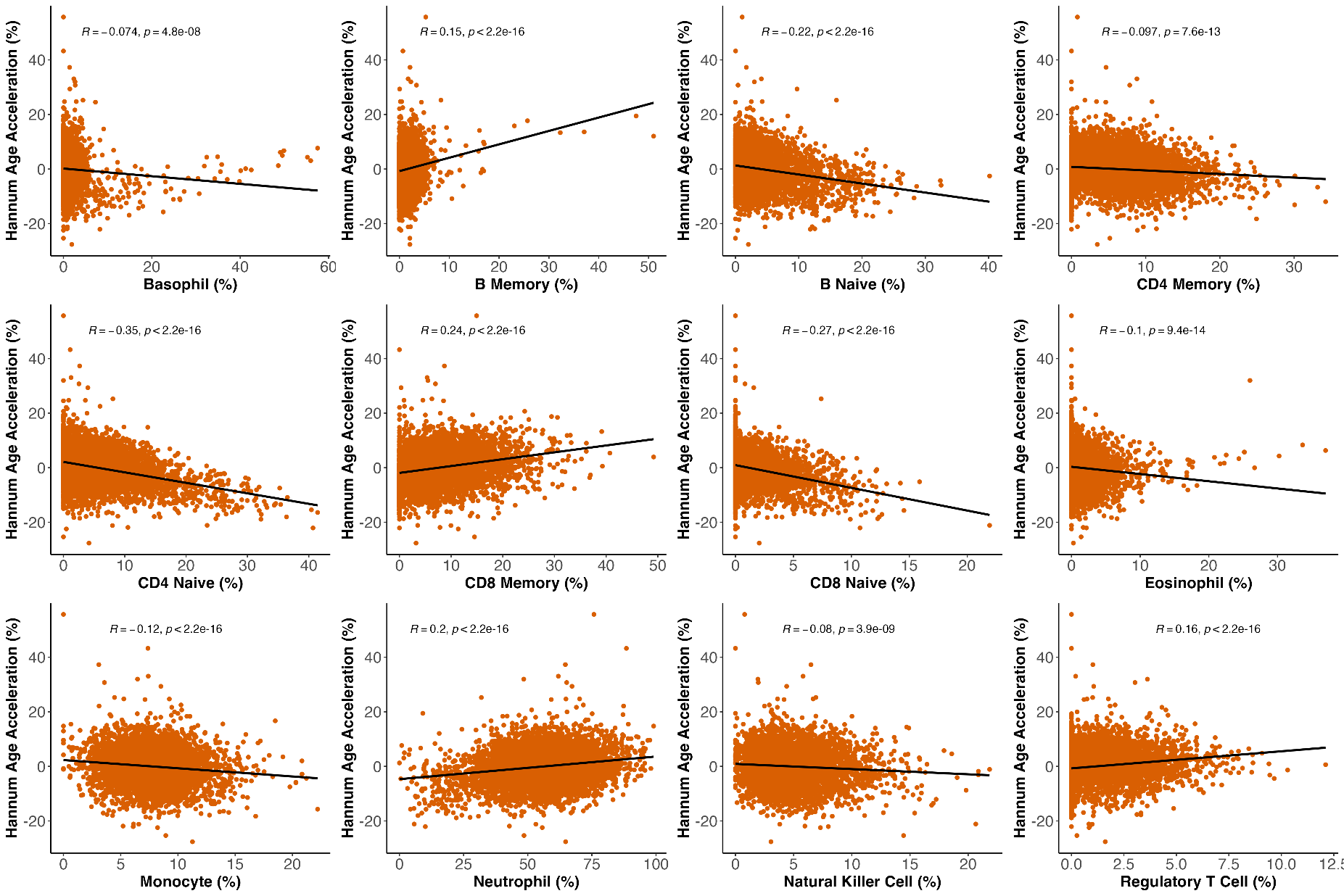


**Supplementary Figure 4.** The correlations between Hannum EAA and immune cell proportions.


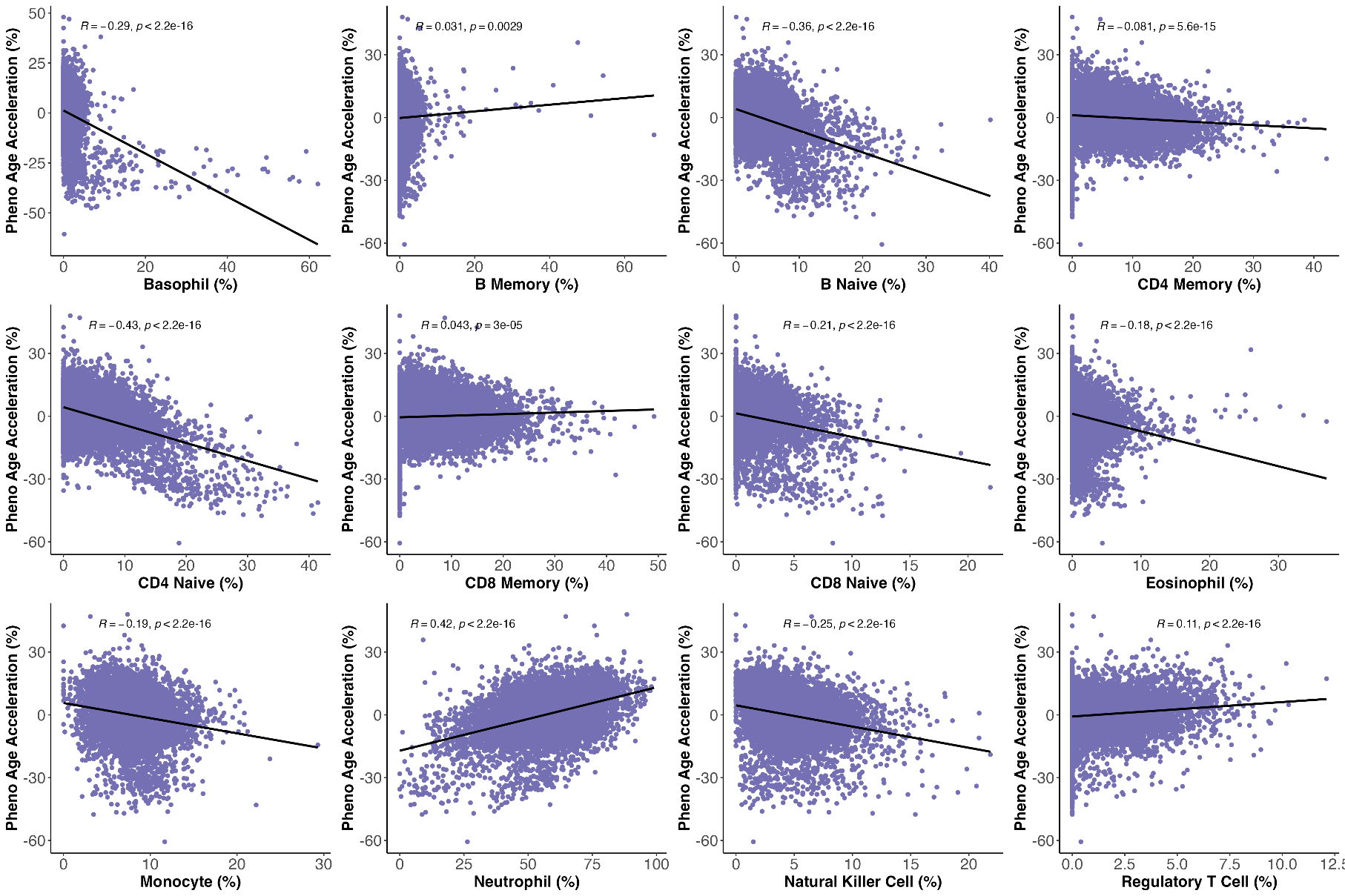


**Supplementary Figure 5.** The correlations between PhenoAge EAA and immune cell proportions.


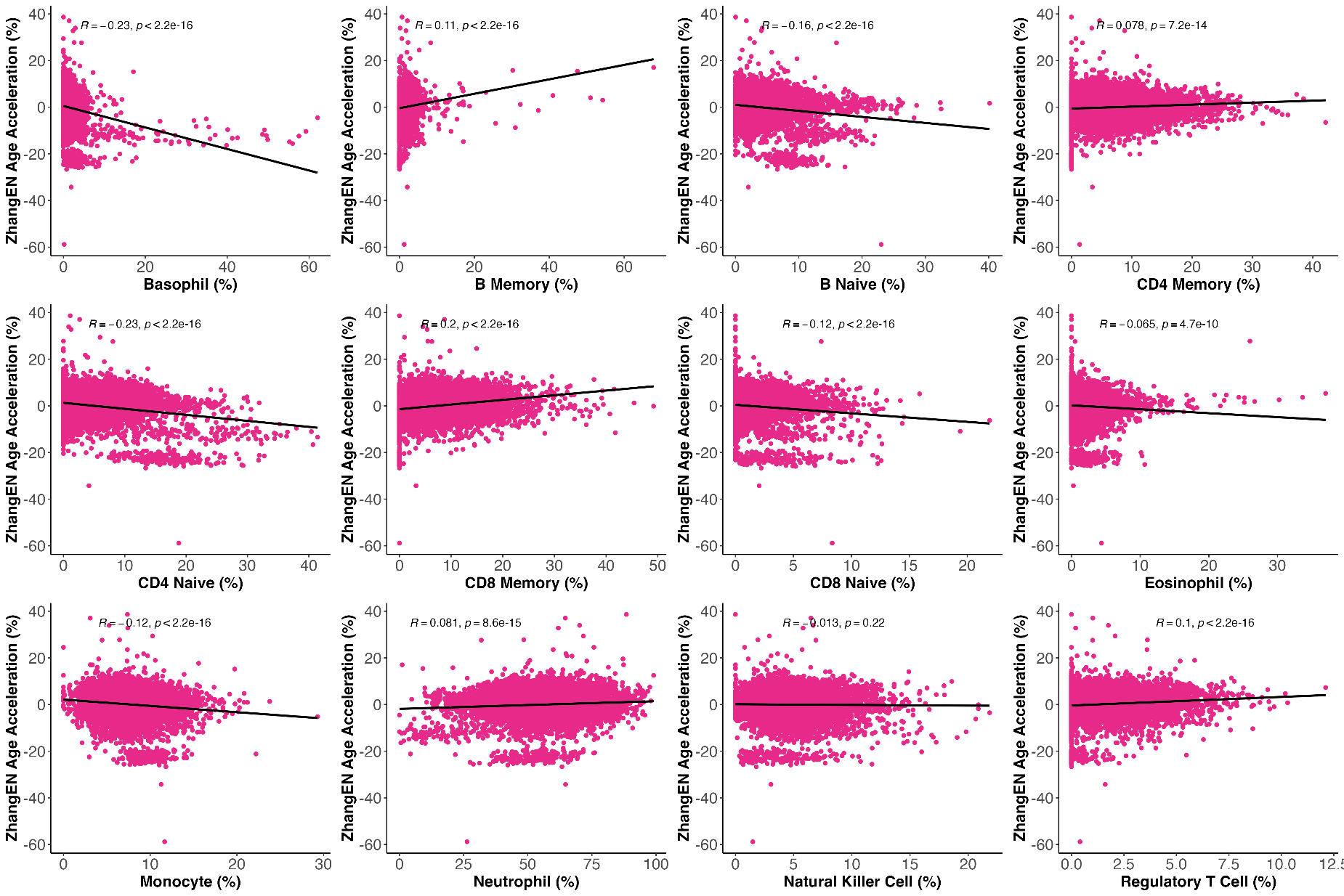


**Supplementary Figure 6.** The correlations between Zhang EAA and immune cell proportions.


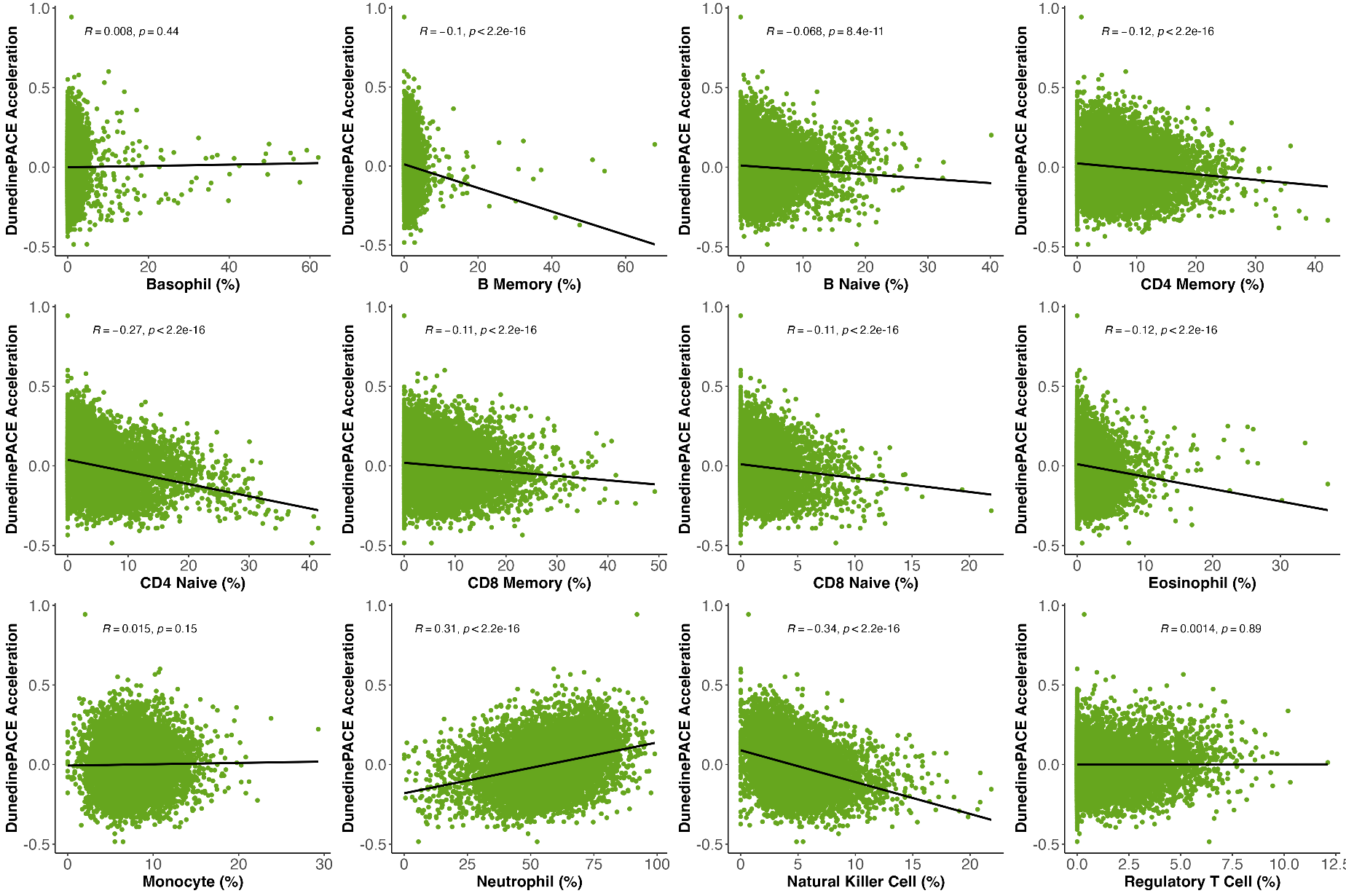


**Supplementary Figure 7**. The correlations between DunedinPACE acceleration and immune cell proportions.


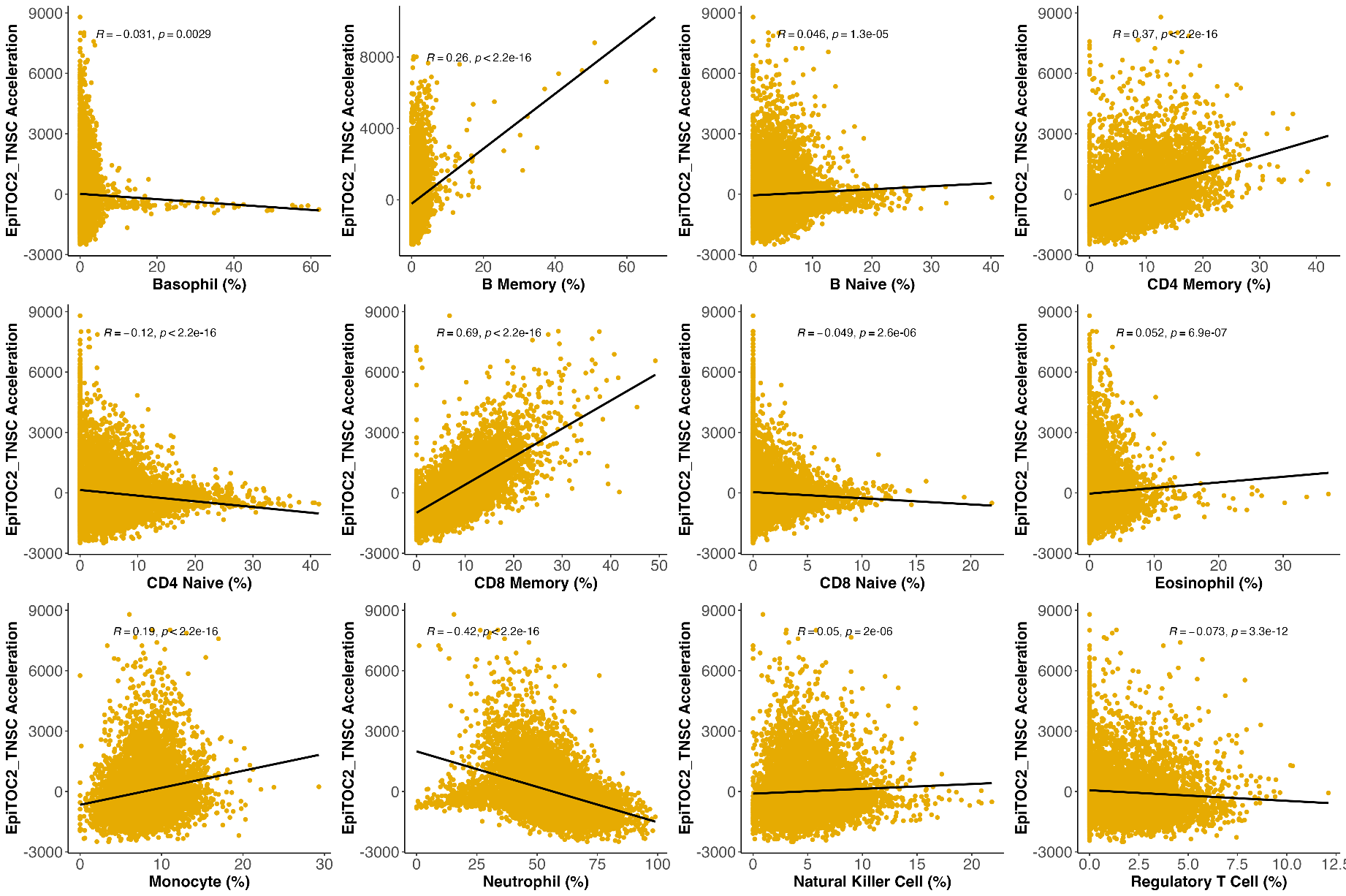


**Supplementary Figure 8.** The correlations between EpiTOC2 TNSC acceleration and immune cell proportions.


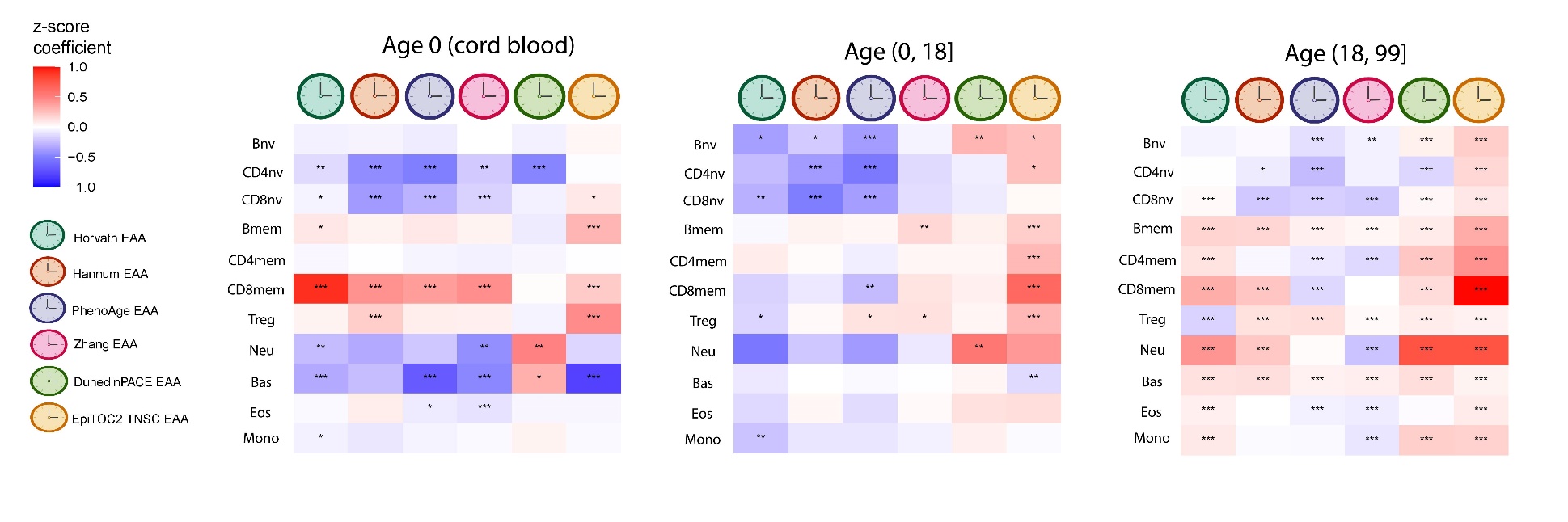


**Supplementary Figure 9.** The associations between individual immune cells and EAA while accounting for the influence of other immune cell proportions (*FDR<0.05, **FDR<0.01, ***FDR<0.001).

.


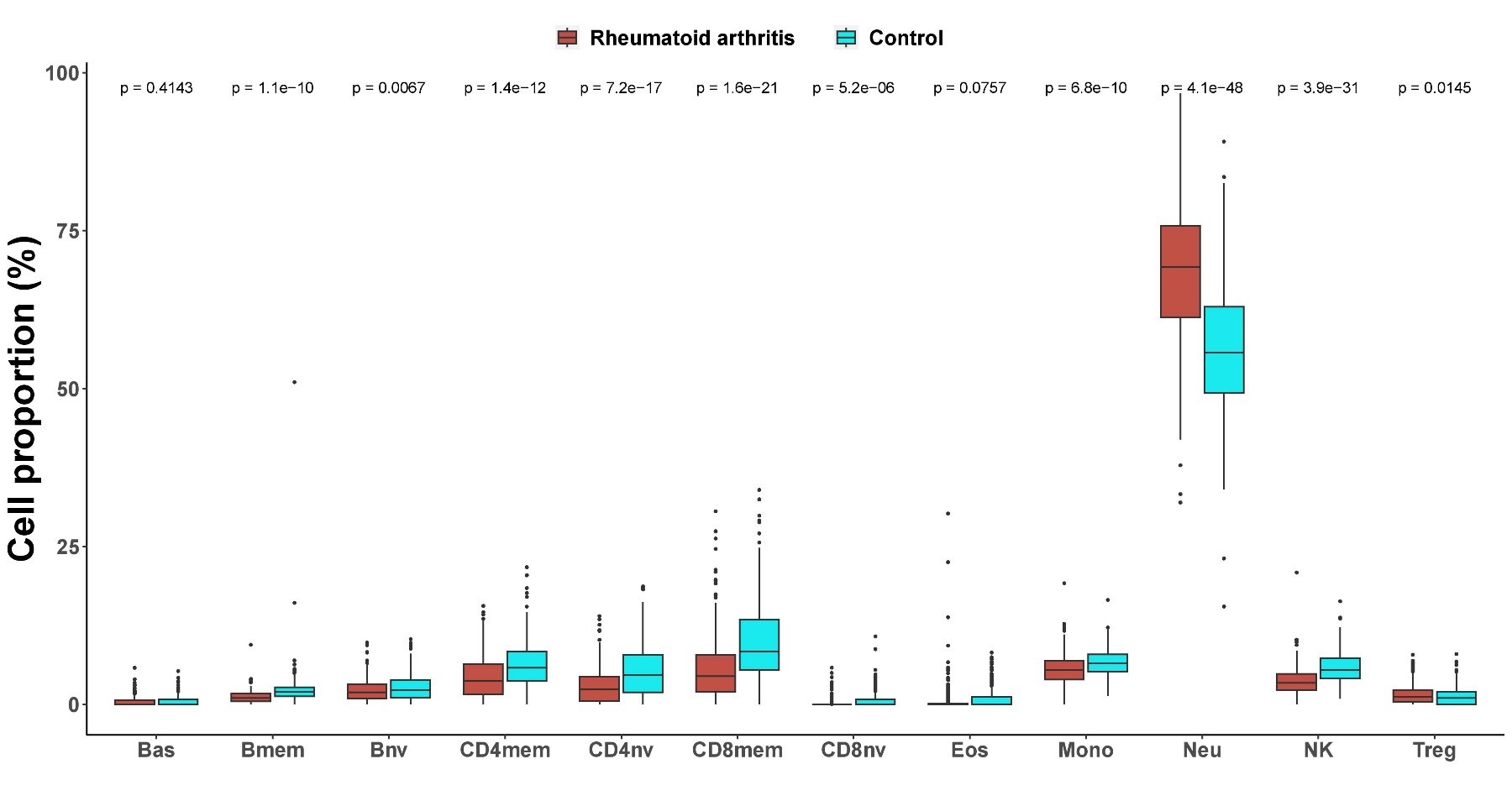
**Supplementary Figure 10.** Immune cell proportion differences between RA cases and controls.


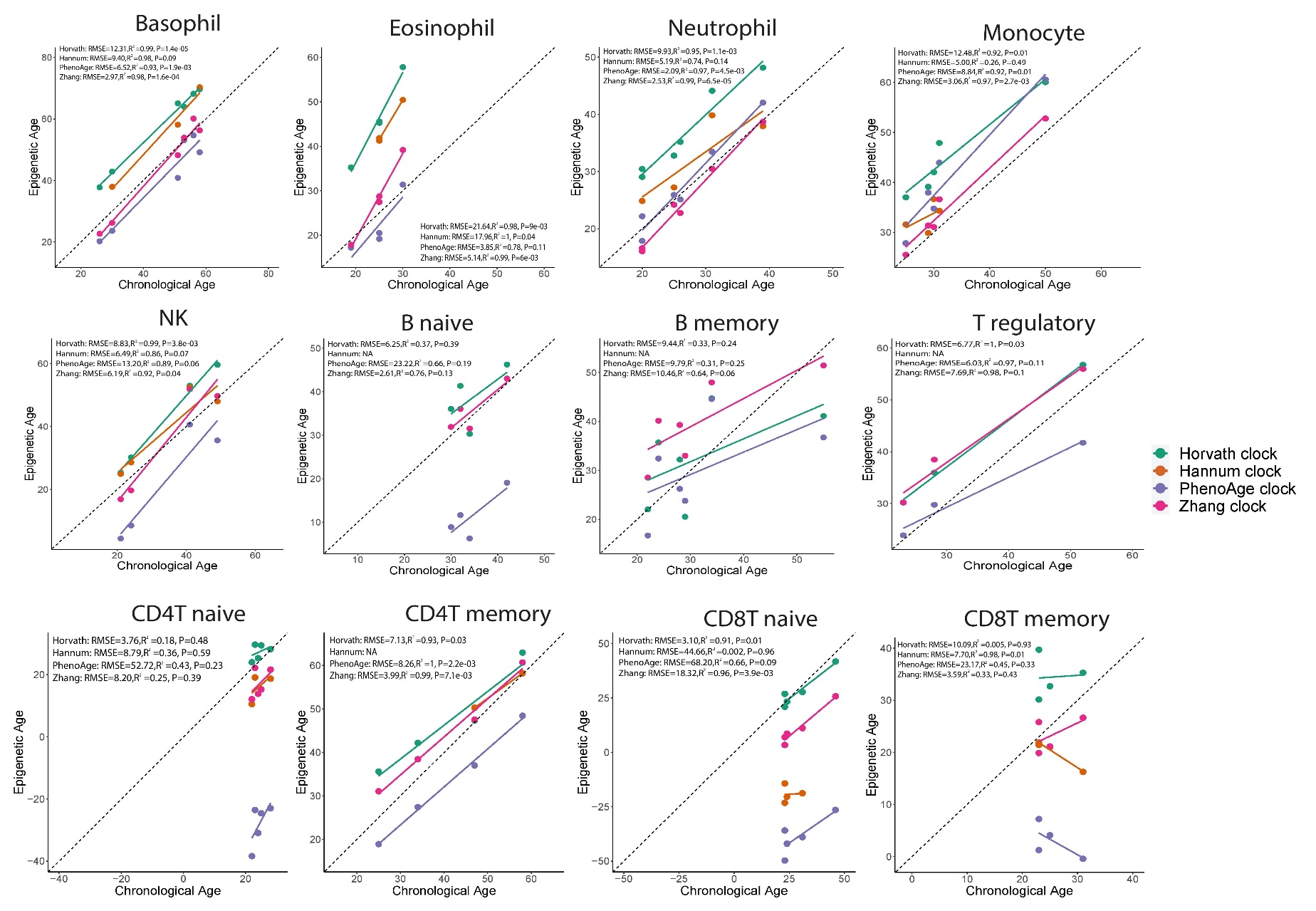


**Supplementary Figure 11.** The performance of Horvath, Hannum, PhenoAge, and Zhang clocks in predicting chronological age using 12 purified immune cell types.


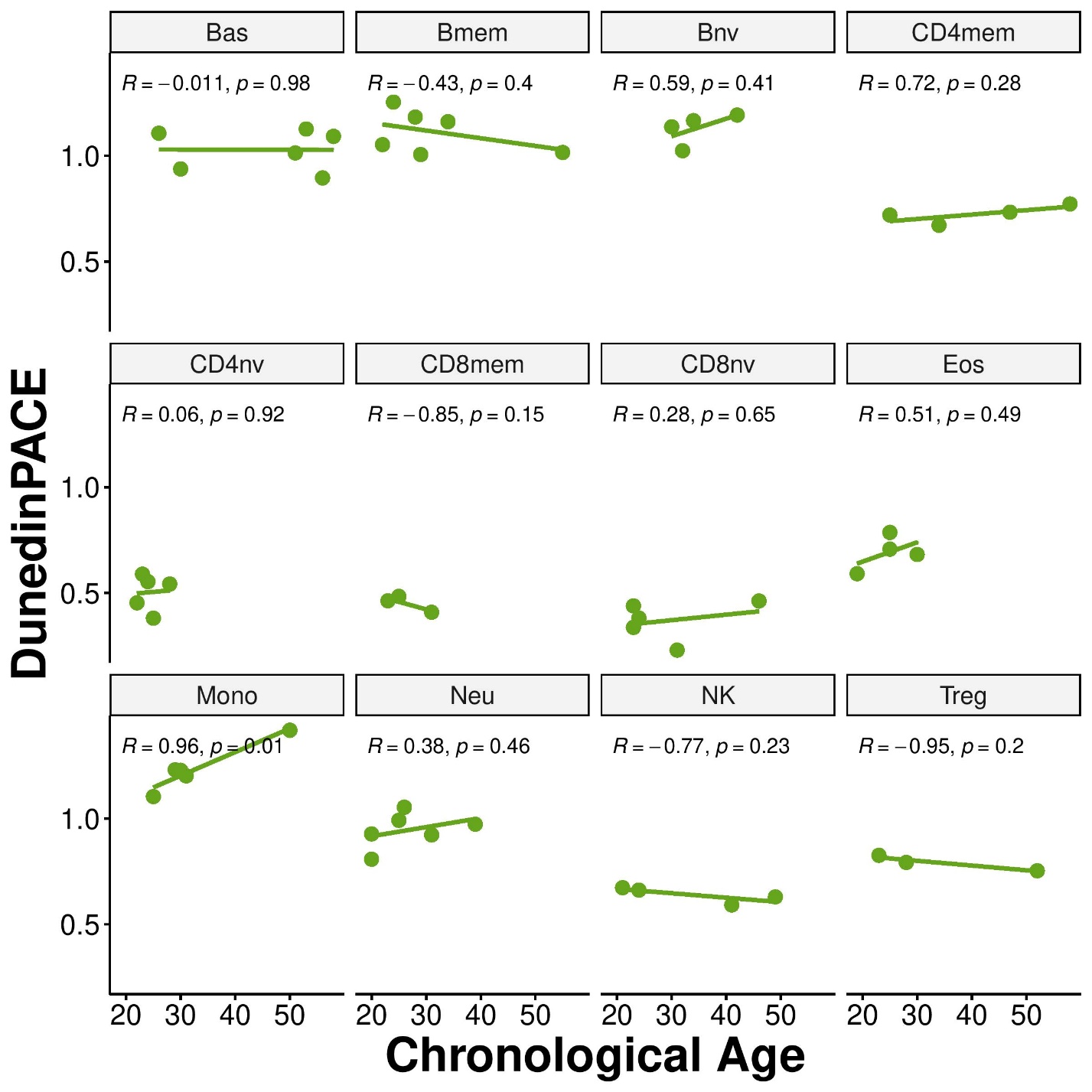


**Supplementary Figure 12.** The correlations between DunedinPACE and chronological age in 12 purified immune cell types.


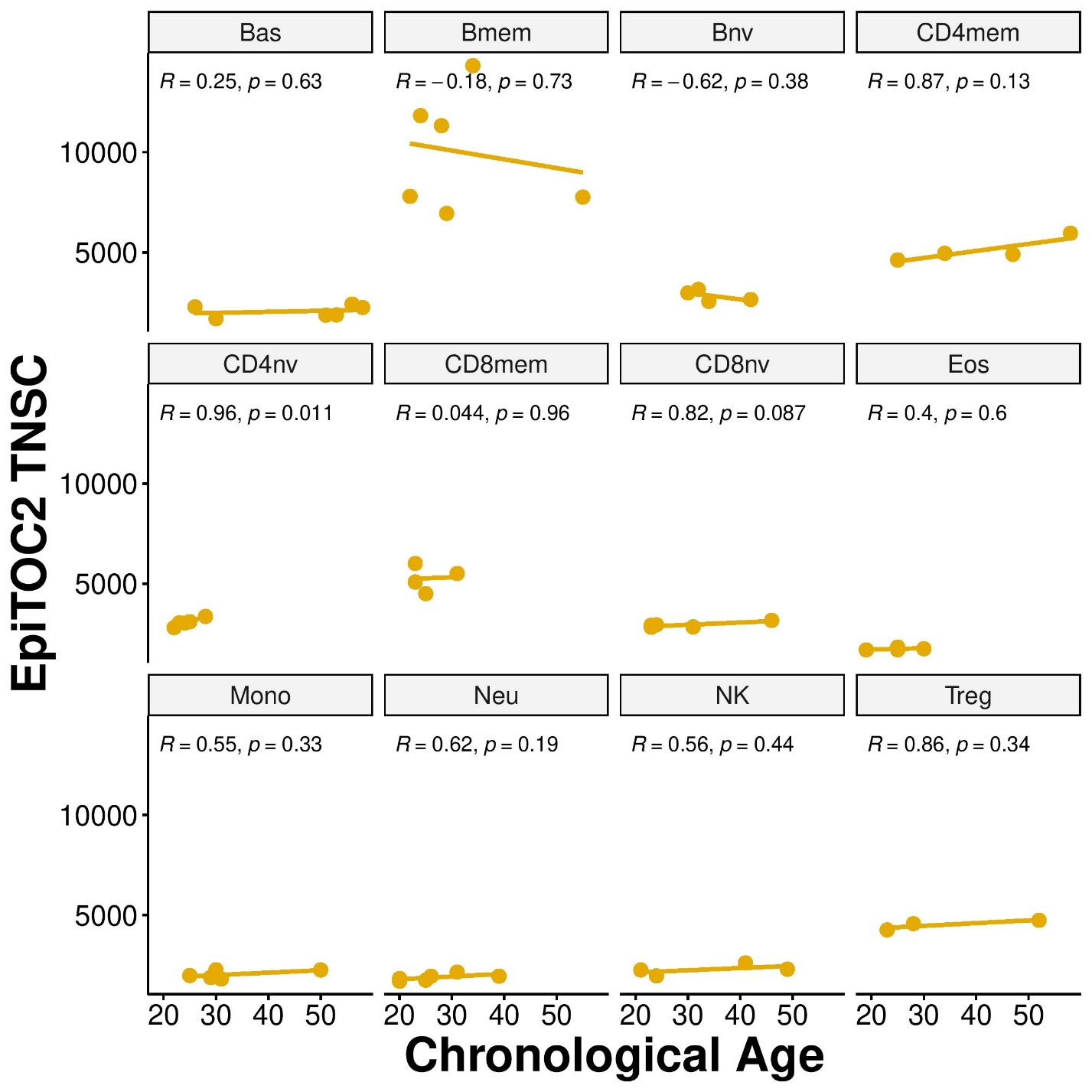


**Supplementary Figure 13.** The correlations between EpiTOC2 TNSC and chronological age in 12 purified immune cell types.


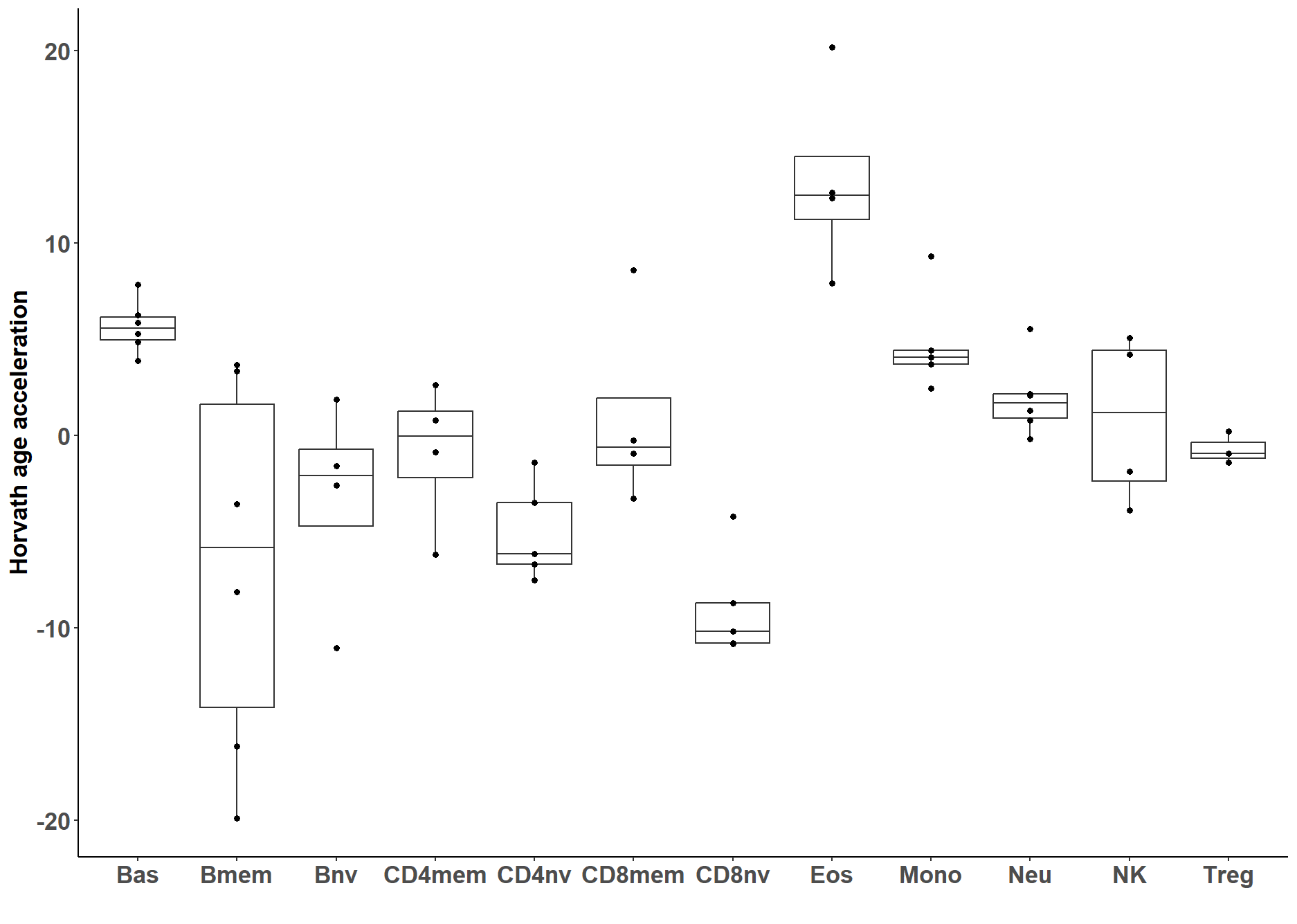


**Supplementary Figure 14.** The distribution of Horvath EAA in purified immune cell types.


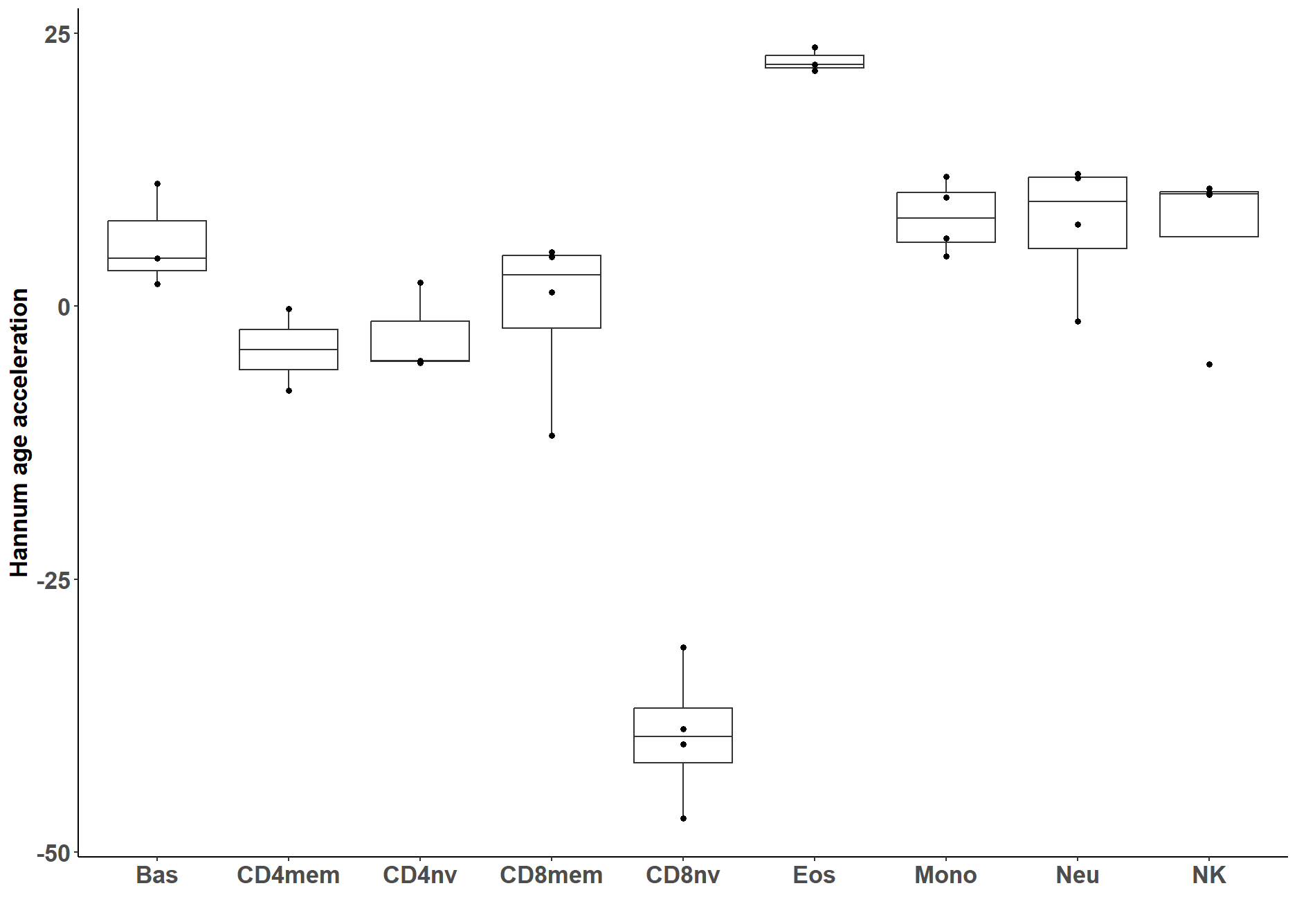


**Supplementary Figure 15.** The distribution of Hannum EAA in purified immune cell types.


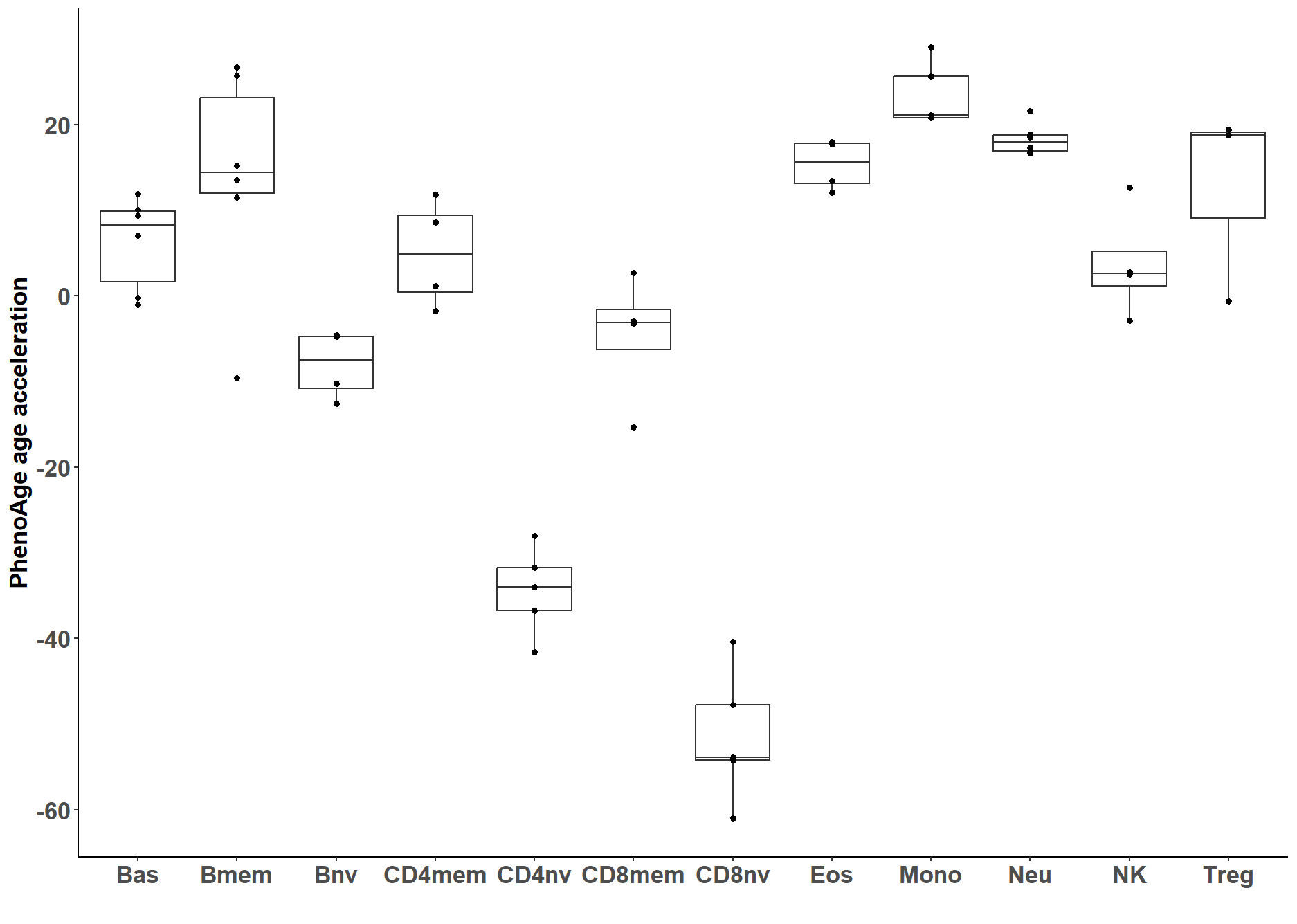


**Supplementary Figure 16.** The distribution of PhenoAge EAA in purified immune cell types.


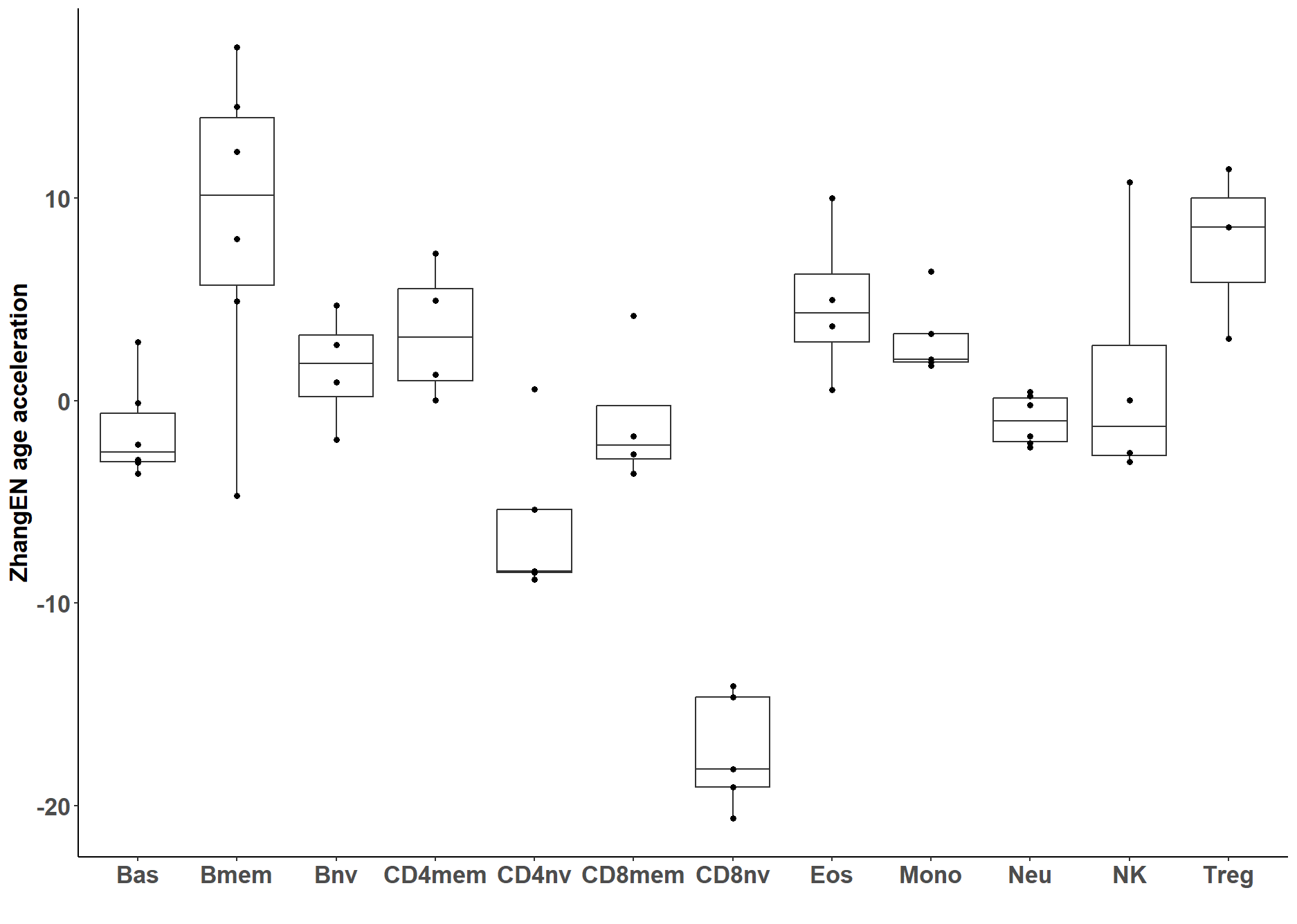


**Supplementary Figure 17.** The distribution of Zhang EAA in purified immune cell types.

**
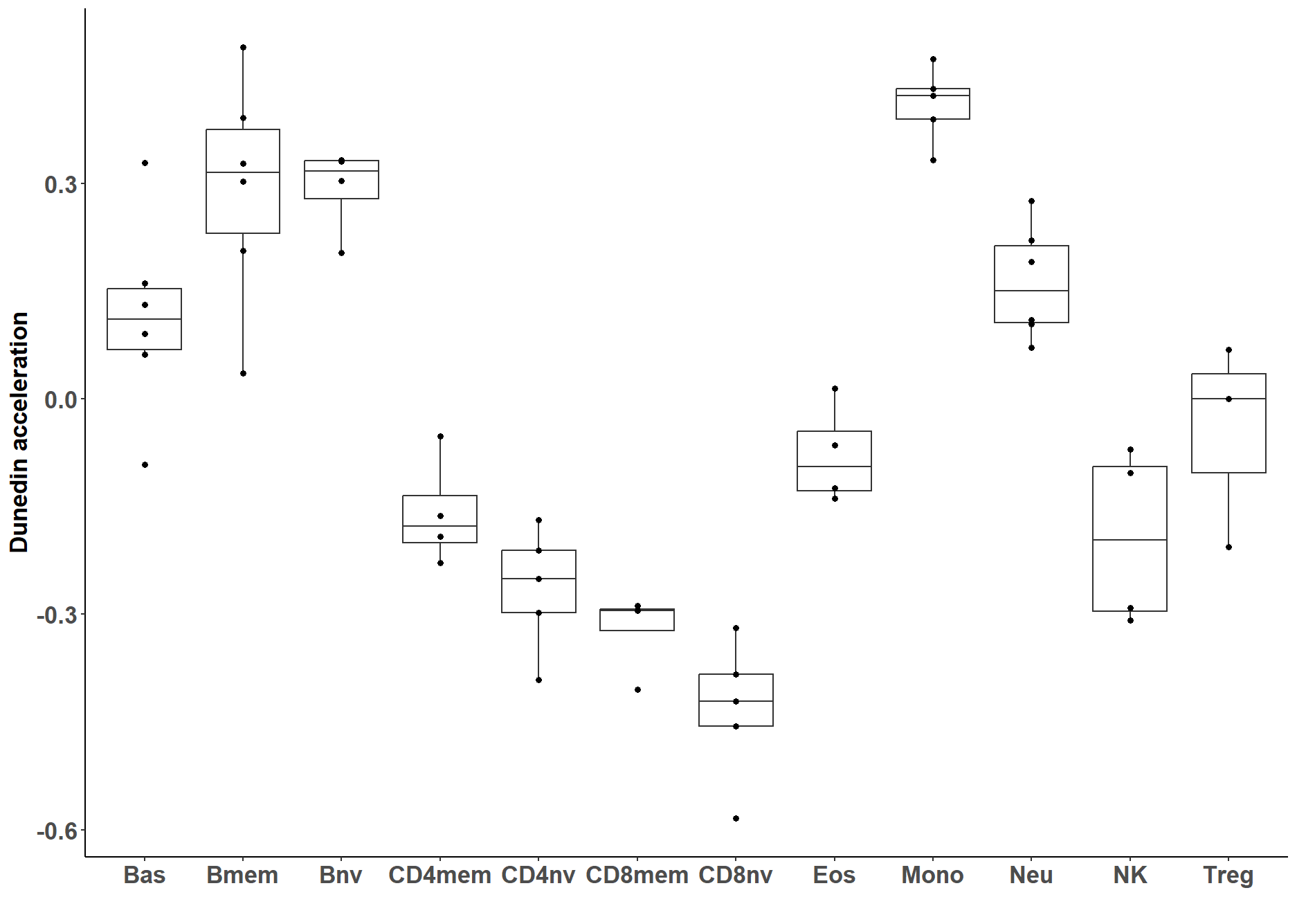
**

**Supplementary Figure 18.** The distribution of DunedinPACE EAA in purified immune cell types.

**
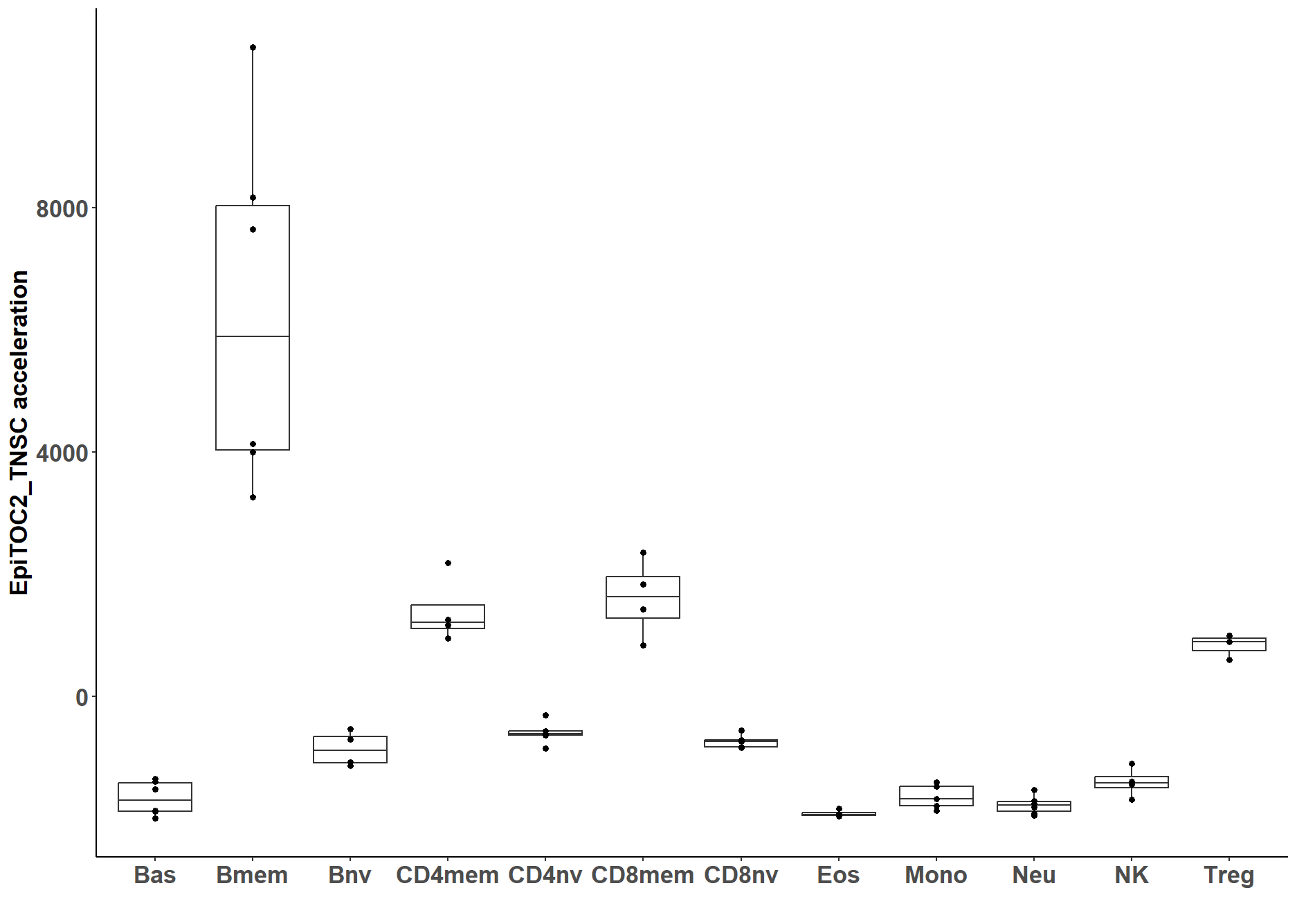
**

**Supplementary Figure 19.** The distribution of EpiTOC2 TNSC EAA in purified immune cell types.


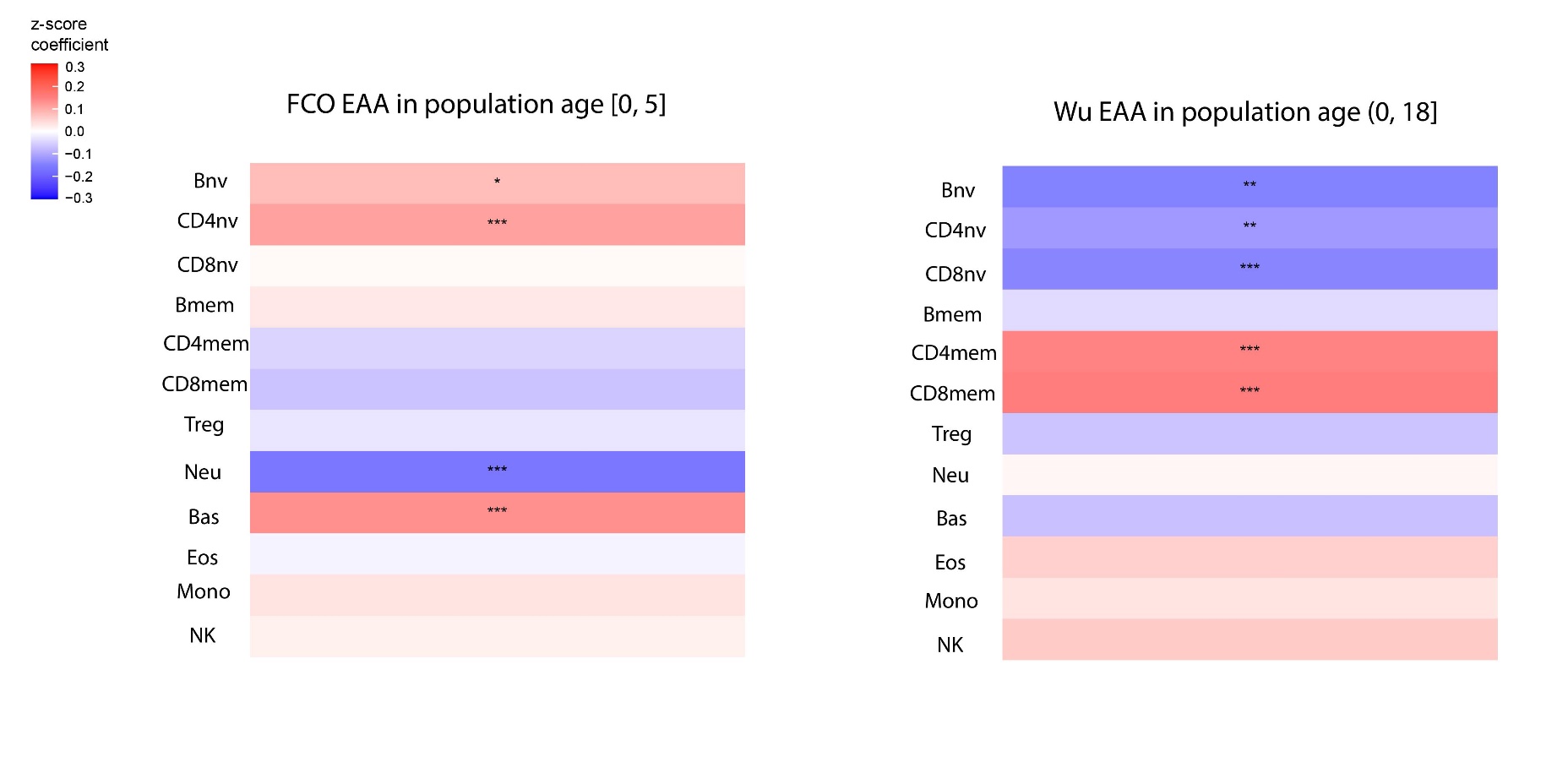
**Supplementary Figure 20.** The association between immune cell composition and pediatric clock EAA after adjusting for chronological age, sex, ancestry, and disease status (*FDR<0.05, **FDR<0.01, ***FDR<0.001).

.


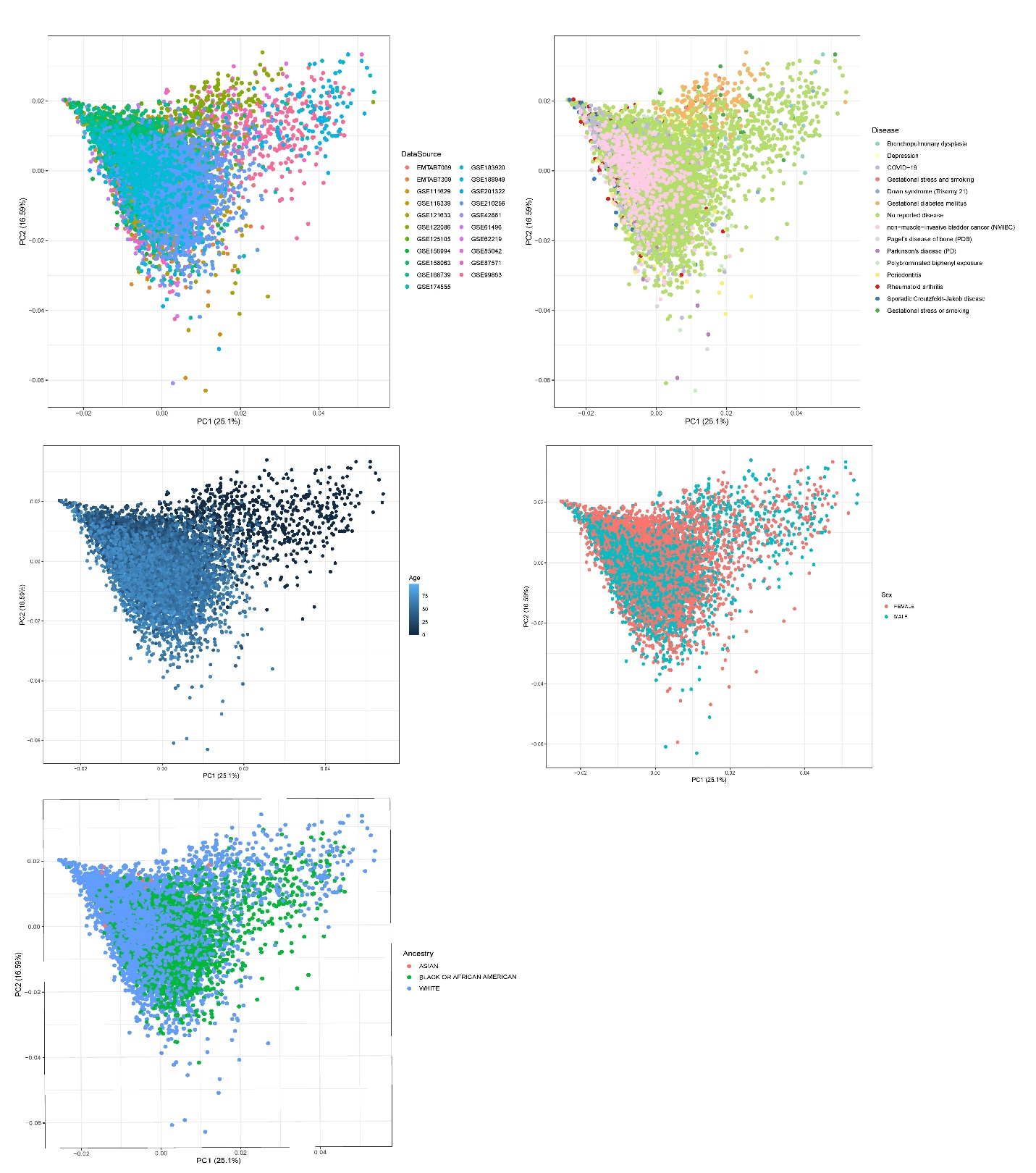


**Supplementary Figure 21.** The relationships between the top principal components (PC1 and PC2) and multiple variables were examined, including data sources (i.e., batch), age, sex, ancestry, and disease.

|  | Rheumatoid arthritis | Control |
| --- | --- | --- |
| N | 354 | 354 |
| Male (%) | 101 (28.5) | 101 (28.5) |
| White (%) | 354 (100.0) | 354 (100.0) |
| Age (mean (SD)) | 51.15 (12.05) | 51.15 (12.05) |

**Supplementary Table 1.** The summary of sex, age, and ancestry matched RA cases and controls.

**Supplementary Table 2.** Purified immune cell samples.
